# VTT-006, an anti-mitotic compound, binds to the Ndc80 complex and suppresses cancer cell growth *in vitro*

**DOI:** 10.18632/oncoscience.549

**Published:** 2021-12-10

**Authors:** Leena J. Laine, Jenni H.E. Mäki-Jouppila, Emma Kutvonen, Pekka Tiikkainen, Thomas K.M. Nyholm, Jerry F. Tien, Neil T. Umbreit, Ville Härmä, Lila Kallio, Trisha N. Davis, Charles L. Asbury, Antti Poso, Gary J. Gorbsky, Marko J. Kallio

**Affiliations:** ^1^VTT Health, VTT Technical Research Centre of Finland Ltd., Otaniemi, Finland; ^2^Turku Bioscience Centre, University of Turku and Åbo Akademi University, Turku 20520, Finland; ^3^Drug Research Doctoral Programme, University of Turku, Finland; ^4^Department of Pharmacology, Drug Development and Therapeutics, University of Turku, Turku, Finland; ^5^Department of Biosciences, Åbo Akademi University, Turku, Finland; ^6^Department of Biochemistry, University of Washington, Seattle, WA, USA; ^7^Department of Physiology and Biophysics, University of Washington, Seattle, WA, USA; ^8^School of Pharmacy, University of Eastern Finland, Kuopio, Finland; ^9^Cell Cycle and Cancer Biology Research Program, Oklahoma Medical Research Foundation, Oklahoma City, OK, USA; ^*^These authors contributed equally to this work

**Keywords:** Hec1, Ndc80, mitosis, cell division, spindle assembly checkpoint

## Abstract

Hec1 (Highly expressed in cancer 1) resides in the outer kinetochore where it works to facilitate proper kinetochore-microtubule interactions during mitosis. Hec1 is overexpressed in various cancers and its expression shows correlation with high tumour grade and poor patient prognosis. Chemical perturbation of Hec1 is anticipated to impair kinetochore-microtubule binding, activate the spindle assembly checkpoint (spindle checkpoint) and thereby suppress cell proliferation. In this study, we performed high-throughput screen to identify novel small molecules that target the Hec1 calponin homology domain (CHD), which is needed for normal microtubule attachments. 4 million compounds were first virtually fitted against the CHD, and the best hit molecules were evaluated *in vitro*. These approaches led to the identification of VTT-006, a 1,2-disubstituted-tetrahydro-beta-carboline derivative, which showed binding to recombinant Ndc80 complex and modulated Hec1 association with microtubules *in vitro*. VTT-006 treatment resulted in chromosome congression defects, reduced chromosome oscillations and induced loss of inter-kinetochore tension. Cells remained arrested in mitosis with an active spindle checkpoint for several hours before undergoing cell death. VTT-006 suppressed the growth of several cancer cell lines and enhanced the sensitivity of HeLa cells to Taxol. Our findings propose that VTT-006 is a potential anti-mitotic compound that disrupts M phase, impairs kinetochore-microtubule interactions, and activates the spindle checkpoint.

## INTRODUCTION

Highly expressed in cancer 1 (Hec1) is a conserved mitotic protein needed for faithful chromosome segregation and the maintenance of genomic balance [1, 2]. Together with Nuf2, Spc24, and Spc25, Hec1 forms a dumbbell-shaped heterotetramer called the Ndc80 complex that stably locates to the outer kinetochore plate of chromosomes throughout mitosis [1–3]. In the complex, the globular domains and N-terminal tails of the Hec1-Nuf2 dimer face outwards towards microtubules and the Spc24-Spc25 dimer orients towards the centromere [2]. The Ndc80 complex is a part of a large kinetochore signaling network termed KMN, which consists of the KNL-1 protein and the Mis12 protein complex [4]. The Ndc80 complex facilitates proper kinetochore-microtubule attachments [1, 5] and chromosome congression to the spindle equator [6], and recruits the spindle assembly checkpoint (the spindle checkpoint) proteins to the kinetochore [7, 8]. During the establishment of correct kinetochore-microtubule interactions, the 80 amino acid long unstructured N-terminal tail of Hec1 and the calponin homology domains (CHDs) of Hec1 and Nuf2 work in concert to control Ndc80 complex binding affinity to microtubules [9–13]. Binding is cooperative and predominantly electrostatic involving positive charges in the CHD and the N-terminal tail of Hec1 and negative charges in the C-terminal tails of tubulin [11]. Aurora B phosphorylates the N-terminal tail of Hec1 at multiple sites and controls the establishment of correct kinetochore-microtubule attachments [14, 15] whereas Nek2 phosphorylates Ser165 in the CH domain and this way controls Hec1 mediated spindle checkpoint signalling and chromosome alignment process [16, 17]. Furthermore, Mps1 phosphorylation sites have been shown to control spindle checkpoint signalling [18].

Because of the important roles of the Ndc80 complex in mitosis and the overexpression of Hec1 in human tumors, targeting the complex may possess therapeutic value in the treatment of cancer. Ndc80 depletion by RNAi has been shown to cause mitotic delay, persistent activation of the spindle checkpoint and subsequent cell death, and in nude mouse xenografts, suppression of tumor growth [7, 19, 20]. Similarly, amino acid mutations or deletions in Hec1 domains involved in microtubule attachment impair the complex’s ability to bind microtubules leading to mitotic errors [9]. Especially in the context of breast cancer, increased Hec1 expression has been suggested to play roles in both pre-neoplastic processes and late tumorigenesis. Hec1 is the most strongly upregulated gene in early breast tumorigenesis (i.e. the transition from normal breast tissue to benign breast tumors and ductal carcinoma *in situ*) and also showed marked upregulation, together with its regulators Aurora B kinases and Nek2, during breast tumor progression from invasive ductal grade I to grade III tumours [21]. A chemical inhibitor INH1 and its analogs have been reported to target Hec1/Nek2 interaction leading to mitotic errors and apoptosis [22–24]. A previous study designed to identify small molecules that target the Hec1-microtubule interface identified SM15. However, this compound was found to hyperstabilize microtubules in the absence of Hec1, causing microtubule stabilization in both interphase and mitotic cells [25]. Here we report the discovery and characteristics of a novel anti-mitotic compound VTT-006, a 1,2-disubstituted-tetrahydro-beta-carboline derivative, that interferes with Hec1-microtubule interaction *in vitro* and perturbs normal mitosis leading to growth suppression in cultured cancer cells.

## RESULTS

### Identification of VTT-006 as a putative anti-Hec1 compound

**Figure 1:**
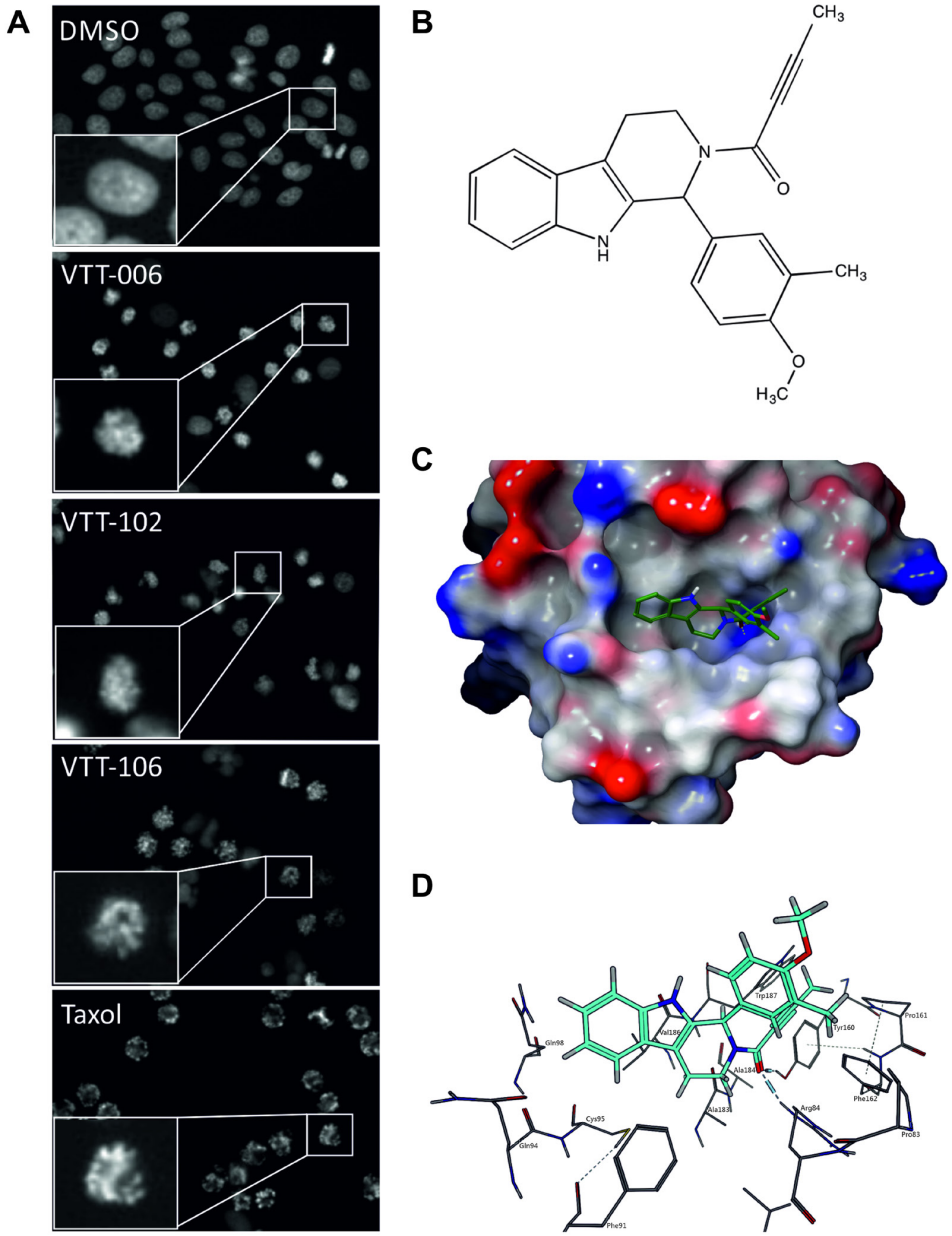
Identification of a novel LMW compound named VTT-006 from virtual and cell-based screens. (**A**) Images of representative wells from the cell-based screen showing mitotic accumulation by VTT-006, -102, -106 and control compound Taxol. HeLa cells stably expressing H2B-GFP were treated with 20 μM concentration of experimental compounds or 600 nM Taxol for 18 h. Insets show magnified images of cells from the populations. (**B**) Structure of the hit compound VTT-006 is shown. (**C**) VTT-006 docked into the microtubule binding pocket of Hec1. Surface view of Hec1 coloured by electrostatic potential is shown. Blue indicates positive charge and red negative charge. Nitrogens are coloured blue and oxygens red in the green stick model of VTT-006. Hydrogen bonds between carbonyl oxygen of VTT-006 and residues Arg84 and Tyr160 are indicated with dashed lines. (**D**) Close-up view illustrates VTT-006 (turquoise) and nearby amino acids of Hec1.

To identify small molecules that interfere with the binding of Hec1 to microtubules, we first performed a virtual *in silico* high-throughput screen (HTS) using the FRED docking software [26]. About 4 million chemical structures obtained from large-diversity compound libraries of different vendors were virtually fitted against the CHD of Hec1 to determine their predicted binding performance. The crystallized structure of Hec1_81–196_ [10] served as the docking template. After consensus scoring and visual inspection of the most potential docking poses, 138 compounds were purchased and tested in cell-based assays for their ability to induce cell cycle arrest at M phase and/or mitotic cell death, which are the reported outcomes of Hec1 loss-of-function by RNAi in cells [7]. The screen was conducted with live HeLa H2B-GFP cells that allowed the visualization of mitotic arrest based on DNA morphology. Three putative lead compounds, named VTT-006, VTT-102 and VTT-106, were identified to possess strong anti-mitotic properties (Figure 1A). Here we report the cellular phenotype and target protein binding properties of VTT-006, 2-(2-butynoyl)-1-(4-methoxy-3-methylphenyl)-2,3,4,9-tetrahydro-1H-beta-carboline (Figure 1B). The compound docks into the CHD of Hec1 (Figure 1C) and is predicted to interact with Hec1 via hydrogen bonds between carbonyl oxygen of VTT-006 and both Arg84 and Tyr160 on Hec1 (Figure 1D, Supplementary Figure 1).

### VTT-006 binds to the recombinant Ndc80 complex and affects its association with microtubules *in vitro*

**Figure 2:**
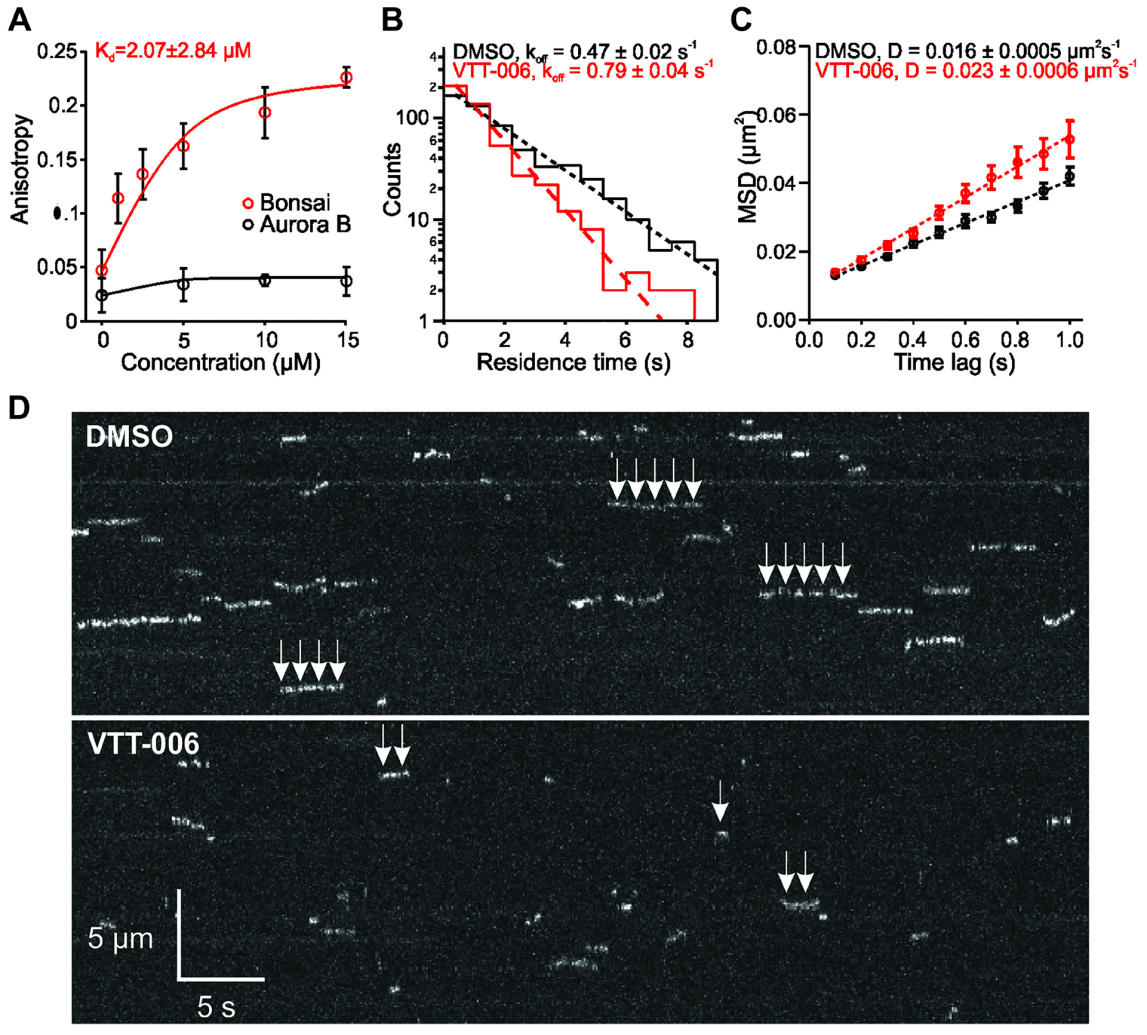
VTT-006 interacts with recombinant Ndc80 complex and reduces the residence time of Ndc80 complex on microtubules *in vitro*. (**A**) Fluorescence polarization assay showing VTT-006 binds to recombinant Ndc80 complex (Bonsai, K_d_ = 2.07 ± 2.84 μM) but not to negative control protein Aurora B. VTT-006 was at 5 μM and protein at 1–15 μM concentration in solution. (**B**) Residence time distributions of Ndc80-GFP on microtubules fit with a single exponential (dashed line) to calculate the off-rate constant, k_off_. (**C**) Mean-squared displacement (MSD) ± SEM versus time lag. A linear fit to the data (dashed line) was used to determine the diffusion constant, D. For (B) and (C), *n* = 581 for DMSO control, *n* = 483 for VTT-006. (**D**) Representative kymographs showing the binding and diffusion of single GFP-tagged Ndc80 complexes (depicted by arrows) on Taxol-stabilized microtubules. Ndc80 complexes were assayed at 6–10 pM in solution, in the absence or presence of 10 μM VTT-006. Position along the microtubule is depicted on the vertical axis over time on the horizontal axis.

In order to validate the *in silico* predicted association of VTT-006 with the Ndc80 complex *in vitro*, we used fluorescence anisotropy to determine the binding constant of VTT-006 to the Ndc80 complex. The recombinant Ndc80 complex (Bonsai), containing the globular domains required for microtubule binding and kinetochore localization, and a shortened rod domain [11], was used as the bait. Results showed that VTT-006 bound to Bonsai but not to negative control Aurora B (Figure 2A). The dissociation constant (K_d_) for VTT-006 was determined using a one site binding model that accounts for ligand depletion and was calculated to be 2.07 ± 2.84 μM. Other hit compounds did not show clear binding to Bonsai using fluorescence anisotropy (results not shown) and were omitted from further studies.

Next, total internal reflection fluorescence *microscopy* (TIRF) was used to study whether VTT-006 affects the binding between the Ndc80 complex and Taxol-stabilized microtubules *in vitro*. Analysis of the binding and diffusion of single Ndc80-GFP complexes on Taxol-stabilized microtubules indicated that VTT-006 perturbed normal association of the complex with microtubules (Figure 2B–2D). The residence time of Ndc80-GFP on microtubules was significantly shorter in the presence of VTT-006 compared to DMSO (Figure 2B) indicating that the compound interferes with Ndc80-microtubule interaction. The off-rate constant increased from 0.47 ± 0.02 s^−1^ to 0.79 ± 0.04 s^−1^ (*p* < 0.001). Furthermore, the diffusion constant increased slightly from 0.016 ± 0.0005 μm^2^s^-1^ to 0.023 ± 0.0006 μm^2^s^-1^ (Figure 2C). Together these results indicate that VTT-006 binds to the Ndc80 complex and affects its association with microtubules *in vitro*.

### VTT-006 induces mitotic arrest and cell death

**Figure 3:**
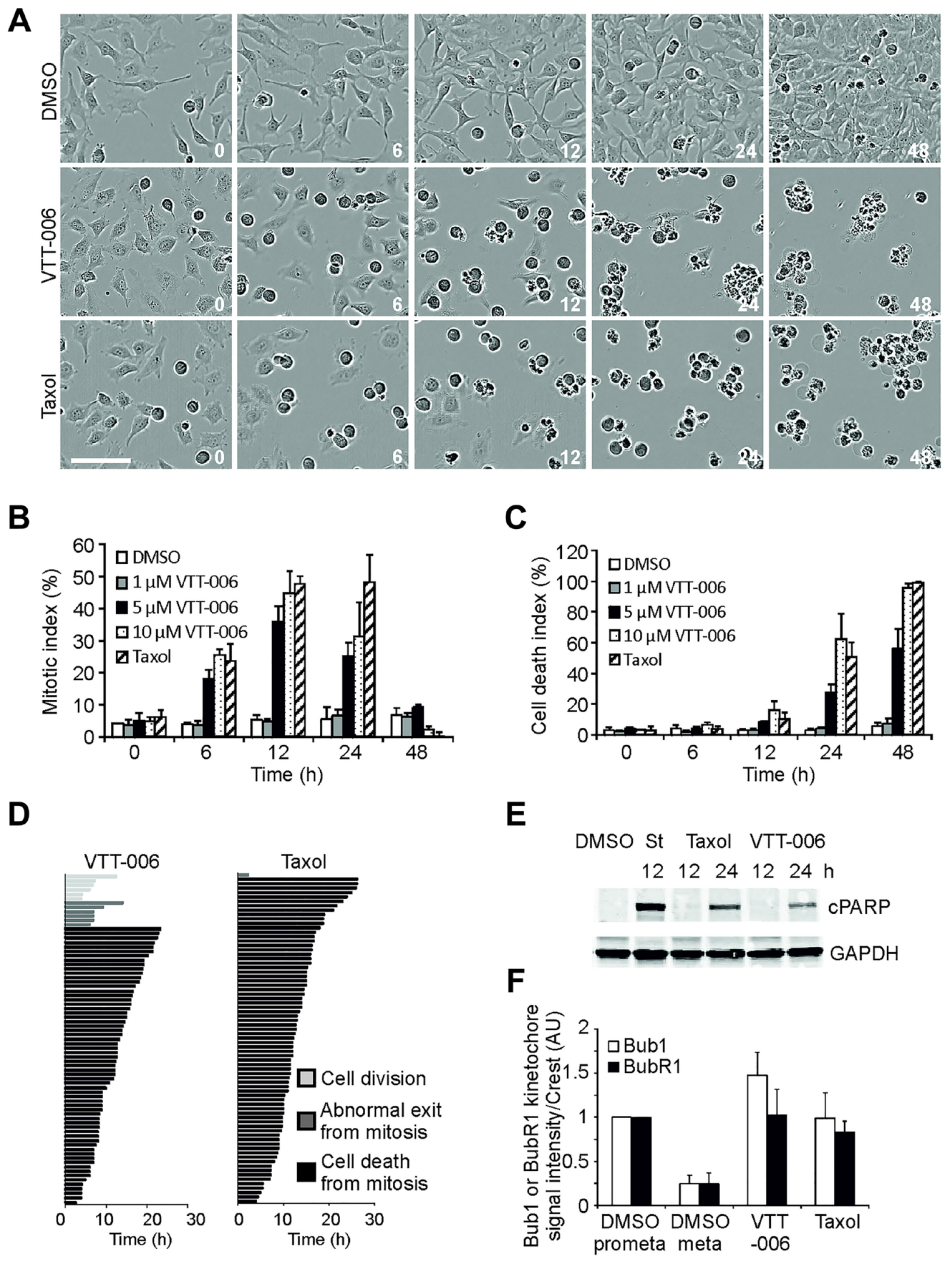
VTT-006 causes mitotic accumulation and apoptosis. (**A**) Representative images from IncuCyte films showing mitotic accumulation followed by cell death upon treatment with VTT-006 or Taxol. Time is indicated in h from the addition of the compounds. (**B**, **C**) Quantification of mitotic (B) and cell death (C) indices from IncuCyte films of cells with indicated compounds and concentrations. Result is average ± SD from 3 replicate experiments. One well (*n* ≥ 150 cells) was analysed in each experiment. (**D**) Analysis of mitotic phenotype and the length of mitosis from IncuCyte films. 75 cells were analysed (25 from each experiment). (**E**) Western blot showing VTT-006 induces PARP cleavage (cPARP) after 24 h treatment. Staurosporine (St) at 1 μM was used as a positive control. (**F**) Quantification of Bub1 and BubR1 kinetochore signals. For VTT-006, only unaligned chromosomes near spindle poles were quantified. Result is average ± SD from 3 replicate experiments (8–15 cells and 20 kinetochores per cell were quantified in each experiment). For (A), (D), (E) and (F) HeLa cells were treated with 10 μM VTT-006 or 100 nM Taxol. Scale bar = 100 μm.

The mitotic effects of VTT-006 were first studied with HeLa cervical cancer cells that were time-lapse monitored using IncuCyte live-cell imager. The compound was applied at different concentrations on actively proliferating cell populations, which were followed for 48 h to determine mitotic and cell death indices. Typically, VTT-006 treated cells accumulated rapidly in mitosis and subsequently underwent cell death (Figure 3A). Cells treated with 5 μM or 10 μM VTT-006 showed significant mitotic accumulation at 6 h (18.0 ± 3.0 and 25.6 ± 1.8%, respectively) and maximal mitotic arrest occurred at 12 h (36.1 ± 4.7% for 5 μM VTT-006 and 44.8 ± 7.1% for 10 μM VTT-006; Figure 3B). Mitotic arrest was followed by cell death culminating at 48 h (56.2 ± 12.8% for 5 μM and 96.0 ± 2.7% for 10 μM VTT-006; Figure 3C). We next analysed the duration of mitotic arrest and fates of individual cells from the IncuCyte films (Figure 3D). Treatment of cells with 10 μM VTT-006 resulted in mitotic arrest that lasted on average 11.5 ± 5.5 h (*n* = 75 cells) and was significantly longer compared to DMSO control (1.2 ± 0.3 h, *n* = 75 cells, *p* ≤ 0.001). The majority of the arrested cells appeared to die directly from mitosis (84.0% of cells), while the rest either divided to produce daughter cells (8.0%) or exited M phase with failed cytokinesis (8.0%). Taxol treated control cells died from mitosis (98.7% of cells) after mitotic arrest had lasted on average 13.1 ± 5.3 h. Western blot analysis of cell extracts prepared from VTT-006 or Taxol treated HeLa cells showed that both compounds caused the appearance of cleaved PARP at 24 h, confirming that the compounds induce apoptosis (Figure 3E).

Mitotic arrest is typically due to persistent spindle checkpoint activity in response to errors in spindle formation and/or kinetochore-microtubule attachments [27]. To find out the spindle checkpoint activity status in DMSO, VTT-006 or Taxol treated cells, we compared the levels of spindle checkpoint proteins, Bub1 and BubR1, at the kinetochores of HeLa cells using immunostaining. Kinetochore localization of these proteins marks active spindle checkpoint signalling [28, 29]. As expected, Bub1 and BubR1 kinetochore signals diminished markedly in DMSO treated control cells when mitosis proceeded from prometaphase to metaphase (Figure 3F, Supplementary Figure 2). In VTT-006 treated cells, Bub1 and BubR1 signals remained high at kinetochores of unaligned chromosomes (Figure 3F, Supplementary Figure 2) suggesting that VTT-006 induced mitotic arrest is spindle checkpoint mediated. We conclude that VTT-006 triggers spindle checkpoint-dependent mitotic arrest that eventually leads to an increase in apoptotic signals.

### Impaired chromosome congression and oscillations in cells treated with VTT-006

**Figure 4:**
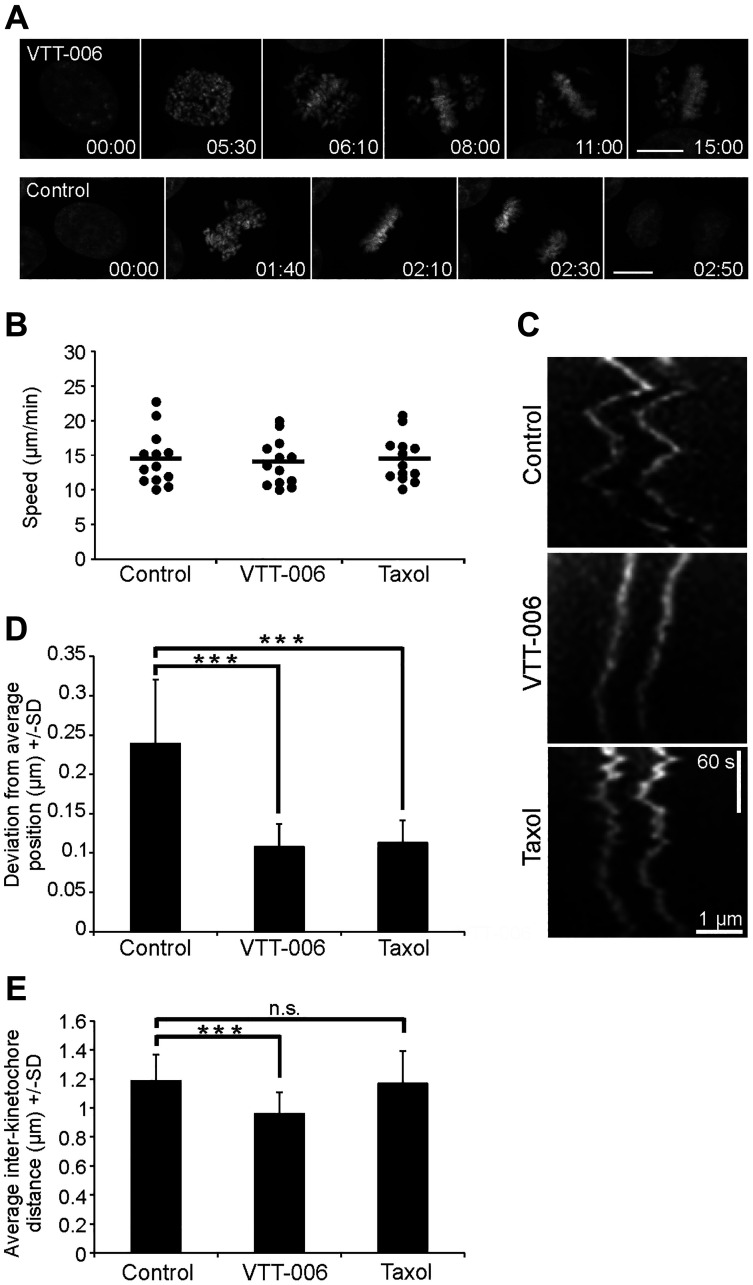
VTT-006 treatment results in chromosome congression defects and dampened kinetochore oscillations at the metaphase plate. (**A**) Still images from time-lapse films of a representative HeLa H2B-GFP cell treated with 5 μM VTT-006, and an untreated control. Time is shown in hours and minutes. (**B**) Graph showing the effect of VTT-006 on rapid (≥10 μm/min) chromosome movements in early mitotic cells. Cells were imaged at NEB with 5 s interval and individual chromosomes/kinetochores were tracked to calculate the average speed of movement. *N* = 13 kinetochores from 6–7 cells. (**C**) Kymographs from representative GFP-Spc24 HeLa cells showing metaphase kinetochore oscillations. (**D**) Graph showing deviation from average kinetochore position ± SD. (**E**) Inter-kinetochore distance in mitotic GFP-Spc24 HeLa cells. Result is average from 85 kinetochore pairs ± SD. (B–E) GFP-Spc24 Hela cells were used to track kinetochores in cells treated with DMSO, 5 μM VTT-006 or 20 nM Tx for 1–3 h. Data were obtained from sister kinetochores that were in the same focal plane.

Mitotic progression was next analysed by high resolution live-cell imaging of HeLa H2B-GFP cells, which were followed for 15 h with 5 min interval after treatment with 5 μM VTT-006. Live cell imaging results indicated that drug treatment caused defects in chromosome congression to the metaphase plate: several chromosomes remained unaligned near spindle poles while most chromosomes congressed to the metaphase plate (Figure 4A; Supplementary Movie 1). Defects in chromosome alignment persisted for several hours and cells remained arrested in mitosis until the end of the filming session, or they died directly from mitosis or soon after aberrant exit from mitosis (Supplementary Movie 1). In contrast to this, control cells divided with normal timing (Supplementary Movie 2).

Chromosome congression to the metaphase plate requires the establishment of lateral connections between kinetochores and microtubules in early mitosis allowing kinetochore sliding on microtubules, followed by the formation of stable end-on attachments, in which microtubules become embedded into the kinetochore [30, 31]. To study the effect of VTT-006 on lateral connections, we tracked kinetochores in live HeLa cells expressing GFP-Spc24 as a kinetochore marker. Cells were imaged at nuclear envelope breakdown (NEB) with 5 s interval and individual chromosomes/kinetochores exhibiting rapid movements were tracked to calculate the average speed of movement. Speeds slower than 10 μm/min were excluded from the measurements. In control cells, the average velocity of movement was 14.4 ± 3.9 μm/min whereas in VTT-006 treated cells velocity was 13.9 ± 3.3 μm/min (Figure 4B). Thus, VTT-006 treatment did not affect rapid movements after NEB. Movements were similarly unaffected in cells treated with control drug Taxol at 20 nM concentration (14.4 ± 3.3 μm/min). These results indicated that VTT-006 does not impair lateral connections and are consistent with earlier findings showing that the Ndc80 complex is not essential for lateral connections [32].

In contrast, the Ndc80 complex plays an essential role in the establishment of proper end-on attachments and is needed for oscillatory movements [15, 33]. The mutations of certain residues in Hec1 and Nuf2 have been shown to influence inter-kinetochore tension reflecting the inability of the cells harbouring mutations to have normal kinetochore-microtubule attachments [9, 34]. We measured oscillations by tracking kinetochore pairs in cells stably expressing GFP-Spc24 and transiently transfected with GFP-NuMA for the visualization of poles. Cells were imaged at 5 s intervals after partial chromosome alignment at the metaphase plate and individual kinetochore pairs were tracked in chromosomes aligned at metaphase by time-lapse. Kinetochore movements were tracked in relation to one of the spindle poles. Kymographs in Figure 4C showing representative kinetochore pairs indicate that cells treated with 5 μM VTT-006 or 20 nM Taxol exhibited reduced oscillations in comparison to DMSO treated control cells. We quantified individual kinetochores to calculate deviation from average position, which is indicative of the amplitude of oscillation. Both VTT-006 and Taxol treatments significantly decreased deviation from average position in comparison to control (Figure 4D). Furthermore, VTT-006 treatment decreased inter-kinetochore distance (Figure 4E) whereas, interestingly, Taxol did not affect inter-kinetochore distance despite its effects on oscillation.

We next analysed whether reduced Hec1 kinetochore levels could explain these observed phenotypes. According to immunofluorescent analysis, Hec1 accumulated at kinetochores to similar levels in cells treated with VTT-006 and in metaphase control cells indicating that the protein could accumulate normally at kinetochores in the presence of the drug (Supplementary Figure 3A and 3B). The Hec1 levels at kinetochores were lower in VTT-006 treated cells compared to the prometaphase control cells. In whole cell lysates, the total Hec1 protein quantities were similar in cells treated with VTT-006, vehicle, Taxol or nocodazole (Supplementary Figure 3C). We also assessed whether kinetochores in VTT-006 treated cells could establish cold-stable attachments with microtubules by lysing Hela cells in cold calcium buffer to deplete non-kinetochore microtubules. After treatment of cells with VTT-006, visible microtubule bundles extended towards chromosomes at the metaphase plate as well as near the spindle poles, which suggests existence of stable kinetochore-microtubule attachments (Supplementary Figure 4). Therefore, the inability of some chromosomes to congress was not likely due to the absence of kinetochore-microtubule connections. Furthermore, we tested the effects of VTT-006 on tubulin polymerization *in vitro*. The results demonstrate that VTT-006 slightly stabilizes tubulin polymerization with high 20 μM concentration (data not shown), which can contribute to the observed cellular phenotypes.

To test whether VTT-006 affects chromosomes that have aligned at the spindle equator (i.e., have established correct kinetochore-microtubule attachments), we took advantage of the proteasome inhibitor MG132, which prevents cells from exiting M phase in a spindle checkpoint -dependent manner while allowing normal chromosome congression to the metaphase plate [35]. Cells were pre-treated with MG132 for 2 h to accumulate metaphase cells, and then the culture medium was supplemented with 10 μM VTT-006 or 20 nM Taxol followed by live-cell imaging. As expected, in MG132 treated cells all chromosomes remained in a metaphase configuration for the whole duration of the filming session (Supplementary Movie 3). In contrast, many chromosomes left the metaphase plate in cells treated with VTT-006 and moved close to one of the spindle poles (Supplementary Movie 4) whereas in cells treated with Taxol a more severe disruption of the metaphase plate was observed (Supplementary Movie 5). Therefore, in addition to inhibitory effects on chromosome alignment in early mitosis, VTT-006 also disrupts normal attachments of chromosomes that have already aligned at the metaphase plate.

### VTT-006 effect on chromosome alignment is reversible

**Figure 5:**
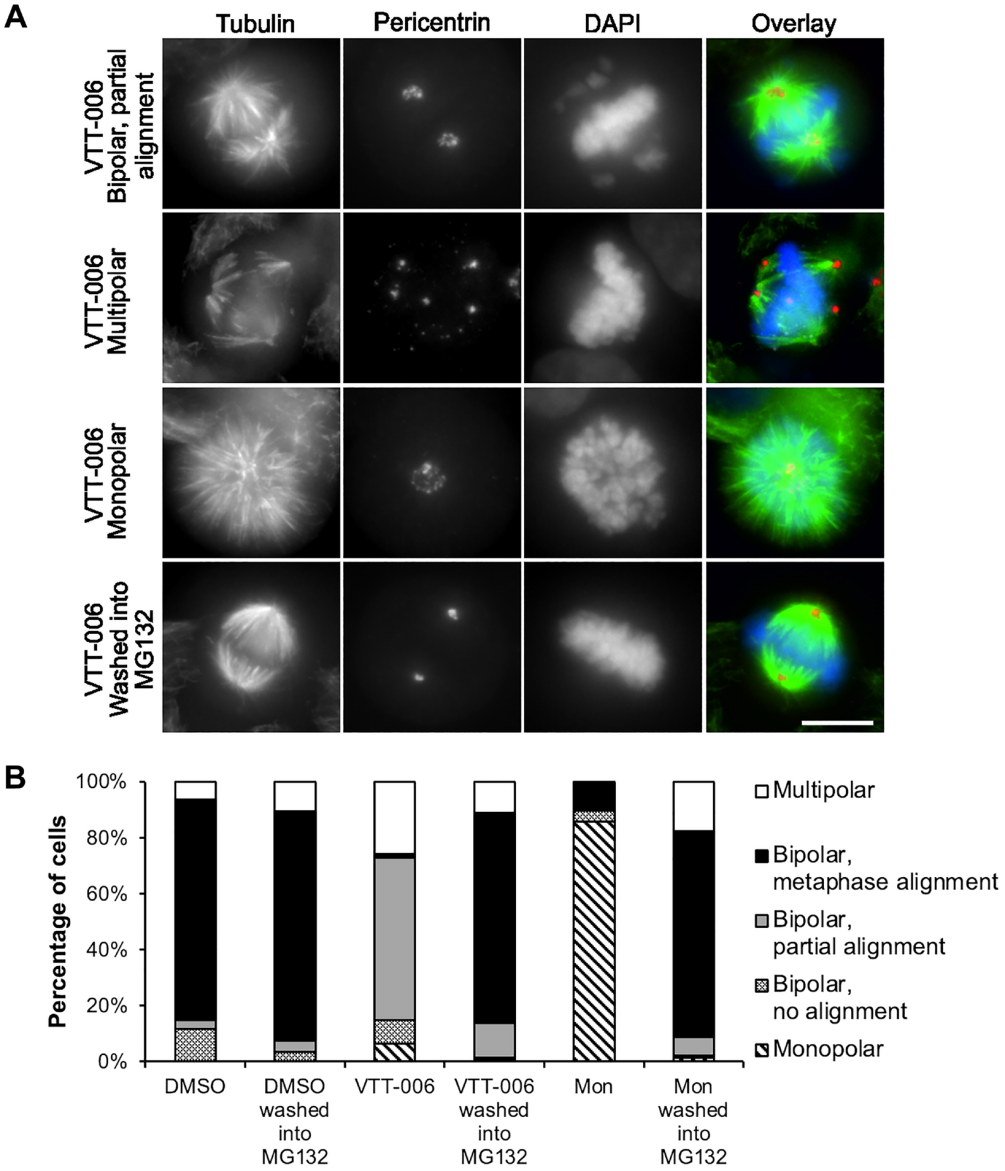
VTT-006 causes reversible chromosome misalignment. (**A**) Images of VTT-006 treated cells displaying chromosome alignment and spindle defects, and a metaphase cell with proper chromosome alignment after drug washout (bottom row). (**B**) Quantification of the phenotype from the drug washout experiment. HeLa cells were treated with DMSO, 10 μM VTT-006 or 100 μM monastrol for 5 h followed by wash into medium containing MG132 for 1.5 h. Result is average from 3 replicate experiments. 100 cells were analyzed in each experiment. Scale bar = 10 μm.

To further quantify the cellular phenotype after treatment with VTT-006 and to assess whether the anti-mitotic effect of the drug is reversible, HeLa cells were treated with DMSO, 10 μM VTT-006 or 100 μM monastrol for 5 h, and processed for immunostainings to detect microtubules, spindle poles and DNA, or alternatively the drugs were washed off and cells were cultured in the presence of MG132 for 1.5 h before fixation and immunostaining. We hypothesized that unless the VTT-006 impact is irreversible, normal chromosome congression to the metaphase plate should ensue upon drug washout and incubation in the presence of MG132. Before washout, analysis showed that VTT-006 treatment resulted in bipolar cells with partial chromosome alignment (58.0 ± 4.1), multipolar cells (25.8 ± 7.2%), bipolar cells with no chromosome alignment (8.4 ± 3.0%), bipolar cells with complete metaphase alignment (1.3 ± 0.6%) and monopolar cells (6.5 ± 5.1%) (Figure 5A and 5B). In sharp contrast to this, after VTT-006 washout, 75.3 ± 5.8% of cells displayed complete metaphase alignment after 1.5 h recovery in the presence of MG132 (Figure 5A and 5B). This metaphase alignment was similar to that seen after monastrol washout into MG132 (73.7 ± 7.9) (Figure 5B). In DMSO treated cell populations, the proportion of complete metaphases was 82.0 ± 1.2% after washout (Figure 5B). These results indicate that VTT-006’s inhibitory effect on chromosome alignment is reversible.

### VTT-006 treatment increases the recruitment of Aurora B kinase at kinetochores

Aurora B kinase has been reported to control Hec1 by modulating its affinity to microtubules [4, 11, 12]. Moreover, the kinase is also required for correction of erroneous kinetochore-microtubule attachments during early mitosis [36]. For these reasons we examined if VTT-006 causes changes in Aurora B localization and activity. Cells were treated with DMSO, VTT-006, Taxol or nocodazole for 6 h, or a combination of Aurora inhibitor ZM447439 and MG132 for 2 h. MG132 was used to block mitotic exit induced by Aurora kinase inhibitor [37]. After treatments, cells were fixed and immunostained with antibodies against Aurora B, p-Aurora B (Thr232) and Crest. Autophosphorylation of Aurora B on Thr232 was measured to detect the active state of the kinase [38]. Analysis revealed a significant accumulation of Aurora B at the centromeres of unaligned chromosomes in cells treated with VTT-006. Indeed, the amount of the kinase was increased by 2.5 –fold in comparison to DMSO controls (Supplementary Figure 5, *p* ≤ 0.05). Moreover, line-scan analysis of the signal distribution across the centromere-kinetochore pairs indicated that Aurora B labelling spread out towards the kinetochores at unaligned chromosomes whereas in control cells Aurora B localized sharply to the inner centromere region (Supplementary Figure 6). The activity of the kinase at unaligned chromosomes was at the level of control prometaphase cells (Supplementary Figure 5). These results indicate that Aurora B accumulates at higher levels at centromeres/kinetochores of unaligned chromosomes in VTT-006 treated cells.

### VTT-006 sensitizes HeLa cells to Taxol

**Figure 6:**
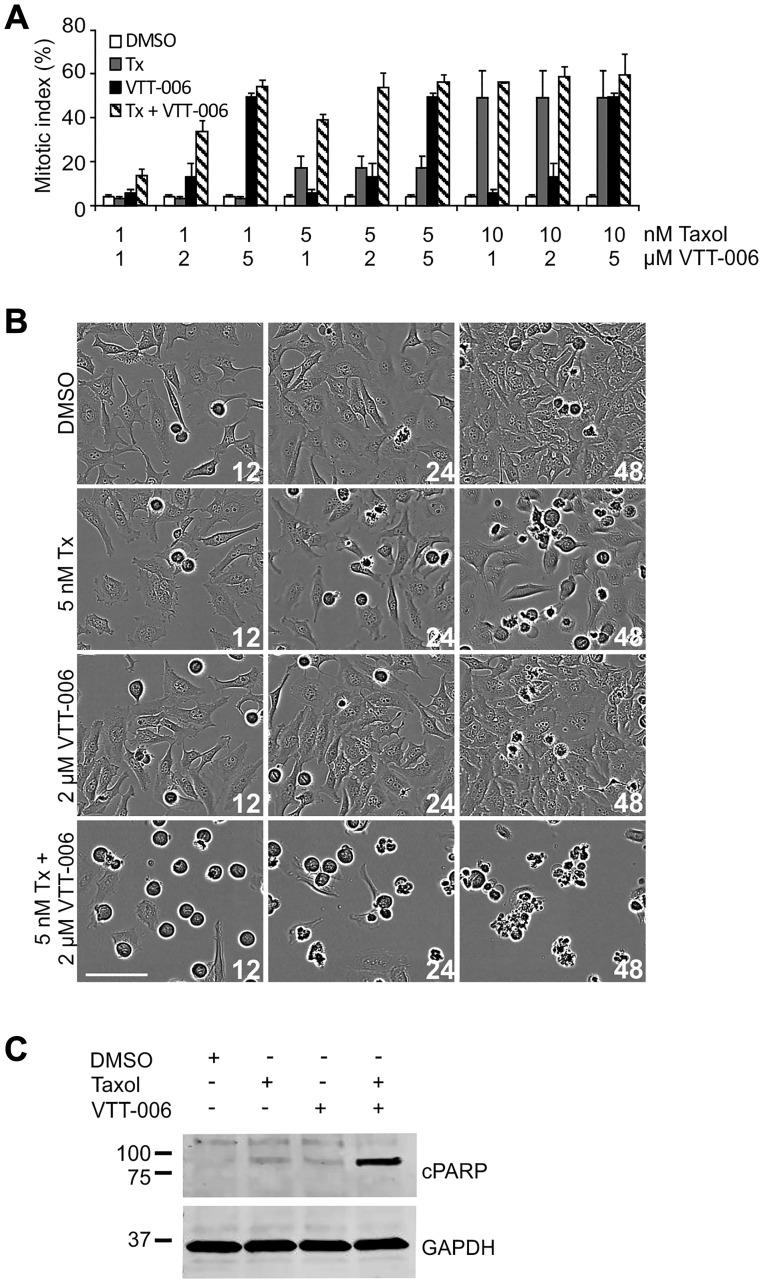
VTT-006 sensitizes HeLa cells to Taxol treatment. (**A**) Quantification of mitotic indices from IncuCyte films using varying concentrations of Taxol and VTT-006. Result is average ± SD from 3 replicate experiments. One well (*n* ≥ 250 cells) was analysed in each experiment. (**B**) Representative images from IncuCyte films showing mitotic accumulation upon treatment with 5 nM Taxol and 2 μM VTT-006 alone or in combination. Time is indicated in h from the addition of the compounds. (**C**) Combination treatment increases cleaved PARP (cPARP) amount compared to single treatments. HeLa cells were treated with 5 nM Tx or 2 μM VTT-006 alone or in combination for 24 h, and collected for Western blot. Scale bar = 100 μm.

Hec1 depletion by RNAi has earlier been shown to cause sensitization to Taxol treatment [39]. To examine possible synergistic effects between VTT-006 and Taxol, we treated HeLa cells with varying concentrations of the two drugs either as single dosages or as drug combinations, and monitored the viability of cells with the IncuCyte imager. Combinations of low concentrations of VTT-006 (1–2 μM) and Taxol (1–5 nM) increased mitotic index in comparison to single drug treatments at the same concentrations (Figure 6A). In some cases, the increases in mitotic index of the combinations appeared to be synergistic rather than simply additive. Cells treated with a combination of 2 μM VTT-006 and 5 nM Taxol exhibited clearly increased cell death 24–48 h after administration of the drug cocktail (Figure 6B) in comparison to cell populations treated with 2 μM VTT-006 or 5 nM Taxol as single treatments. Cell death coincided with the induction of cleaved PARP indicating apoptotic cell death (Figure 6C). Based on these findings, sub-lethal doses of VTT-006 sensitize cells to low nanomolar concentrations of Taxol.

### VTT-006 inhibits the growth of various cancer cell lines

**Figure 7:**
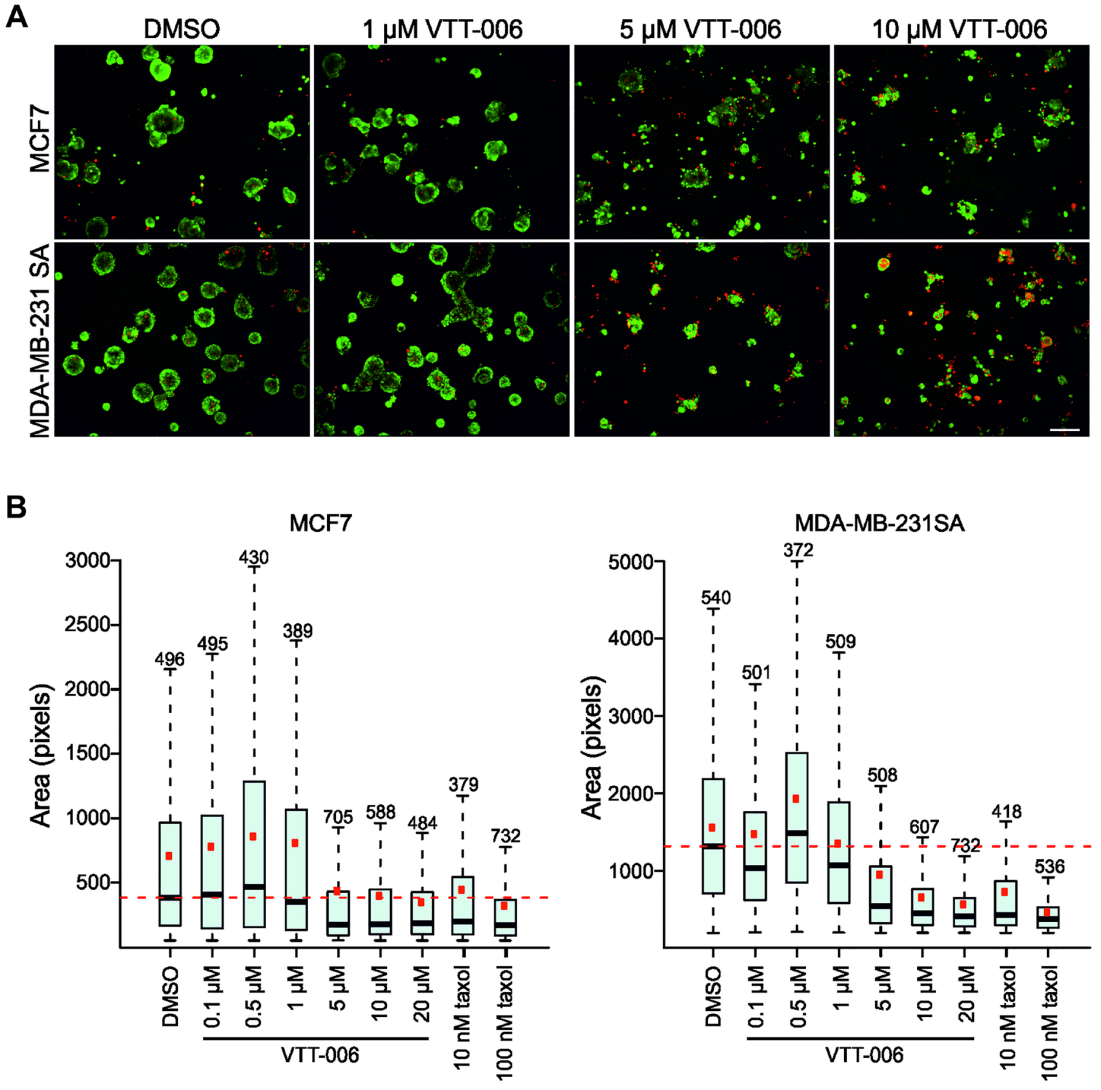
VTT-006 inhibits the growth of breast cancer cell lines in 3D organotypic cell cultures. (**A**) Representative images from 3D cell culture. MCF7 and MDA-MB-231 SA cells were treated with indicated compounds for 5–6 days and stained with calcein (green) to detect live cells and Ethidium homodimer-1 (red) to detect dead cells. (**B**) Quantification of spheroid size. Black line denotes median, red dot mean, box marks first and third quartiles and whiskers show the range of values. Size cut-off value of 50 was used for MCF7 and 200 for MDA-MB-231 SA analysis. Numbers on top of the dotted lines represent the number of spheroids (*n*) analysed from each treatment. Scale bar = 200 μm.

In addition to HeLa cells, the effects of VTT-006 on cell viability were studied on a panel of cell lines including cancer cells derived from breast, prostate, colon, lung and ovarian tissues and three non-malignant cell lines using CellTiter Glo assays. EC_50_ values were in the range of 4.8–11.9 μM for other cell lines except for MCF10A, which had an EC_50_ value of 21.3 μM indicating that this non-tumorigenic breast cell line is more resistant to VTT-006 (Table 1). However, the viability of non-tumorigenic prostate cell lines Ep156T and RWPE-1 was decreased by VTT-006. The same cell lines observed with the IncuCyte imager also showed accumulation in mitosis when treated with 1, 5 or 10 μM VTT-006 with the exception of MCF10A cells, which did not undergo mitotic arrest (results not shown). Finally, growth suppressive effects of VTT-006 were studied in 3D organotypic cell culture system [40] using breast cancer cell lines MCF7 and MDA-MB-231 SA as models. The growth of the multicellular spheroids was efficiently retarded by 5 μM and higher concentrations of VTT-006 determined by morphometric spheroid criteria (Figure 7). The results indicate similar efficacy for VTT-006 in monolayer and 3D cell culture models.

**Table 1:** VTT-006 inhibits the proliferation of various cell lines

Cell line	EC_50_ (μM)
**Non-tumorigenic immortalized cell lines**	
MCF10A (mammary epithelial cells)	21.3 ± 1.5
Ep156T (prostate epithelial cells)	5.7 ± 1.0
RWPE-1 (prostate epithelial cells)	4.8 ± 0.5
**Tumorigenic cell lines**	
HeLa (cervical adenocarcinoma)	6.5 ± 1.3
MCF7 (breast adenocarcinoma)	8.3 ± 1.7
MDA-MB-231 (breast adenocarcinoma)	4.9 ± 1.5
MDA-MB-231-SA (breast adenocarcinoma)	6.7 ± 1.4
LnCaP (prostatic adenocarcinoma)	8.1 ± 2.0
RWPE-2-W99 (tumorigenic derivative of RWPE-1)	6.3 ± 0.6
HCT116 (colorectal adenocarcinoma)	10.4 ± 0.5
A549 (lung adenocarcinoma)	11.9 ± 0.9
Ovcar-3 (ovary adenocarcinoma)	7.5 ± 1.2

Mean EC_50_ values ± SD from 3 replicate experiments are shown.

## DISCUSSION

We describe here the cellular effects and anti-proliferative activities of VTT-006, a small molecule that binds to Hec1 and perturbs its association with microtubules *in vitro*. To our knowledge, no previous studies have reported small molecules that would directly inhibit Hec1 function. One set of developmental compounds originating from the INH1 template has been shown to impair Hec1-Nek2 kinase interaction leading to the degradation of the kinase and growth suppression in multiple human cancer cell lines in culture and in mouse xenografts [22, 24]. In addition, the molecule SM15 has been reported to bind the surface of microtubules independently from Hec1 and to stabilize microtubules and the microtubule-kinetochore interaction [25]. Future comparative studies with these three compounds could further help to determine their different effects on cell proliferation at molecular level. We aimed at the discovery of compounds that would prevent the establishment of proper microtubule-kinetochore interactions via targeting the CHD of Hec1. Perturbation of the dynamic instability of microtubules by filament-destabilizing agents such as vinblastine and vincristine, or by filament-stabilizing drugs paclitaxel, docetaxel and epothilones has been utilised for decades to treat cancer patients [41]. Due to poor cancer cell selectivity of microtubule-targeting drugs and their side effects, such as peripheral neuropathy, new anti-mitotic compounds have been actively developed against e.g., mitotic motor proteins Eg5 and Cenp-E [42, 43], Plk1 and Aurora kinases [44–47], and other key facilitators of cell division [48, 49]. However, the majority of non-microtubule targeting anti-mitotics have not translated their preclinical efficacy to a tolerable clinical response, which has fueled early drug discovery and new target validation studies in the field of mitosis research.

Hec1 is an interesting drug target protein for many reasons. Its normal function is indispensable for ordered chromosome segregation and cell cycle progression [1, 2]. Depletion of the protein by siRNA or virus-mediated RNAi has been shown to suppress cancer cell growth in cells and xenograft models [19, 20]. On the other hand, the oncogenic properties of Hec1 were demonstrated in a transgenic mouse model where protein overexpression was associated with elevated frequency of tumour formation [50]. Importantly, to date Hec1 has not been reported to possess any functions outside mitosis that in principle limits the effects of a Hec1 inhibitor to actively proliferating cell types. Therefore, hypothetically, by interfering with microtubule-kinetochore associations using a Hec1 drug one can avoid the obstruction of microtubule dynamics in non-dividing cells such as neurons and thereby reduce clinical adverse effects in comparison to spindle poisons. These notions together with the well-established Hec1 overexpression found in a wide range of human tumours emphasize the clinical potency of Hec1 inhibition.

Anisotropy measurements indicated clear binding, albeit at low micromolar affinity, for VTT-006 to recombinant Ndc80 complex. This ranks the compound into the group of so-called general-affinity ligands that typically exhibit K_d_ values in the range of 10^-4^ to 10^-6^ M and may bind to more than one protein [51]. Therefore, we cannot exclude the possibility that VTT-006 would target other proteins besides Hec1/Ndc80 complex. The binding data proposes an intermediate affinity for VTT-006 on Hec1, which may enable compound to switch between bound and non-bound states with its ligand(s).

Our results with VTT-006 from *in vitro* and cell-based assays are consistent with a partial inhibition of Hec1/Ndc80 function. TIRF microscopy revealed that the compound significantly shortened the residence time of Ndc80-GFP on Taxol-stabilized microtubules but did not fully abolish the complex’s ability to bind to the filaments. This effect resembled the outcome of mutating Aurora B target residues in the N-terminal tail or complete loss of the tail region, both of which reduce the residence time of Ncd80 complex on microtubules in TIRF assays [52]. Partial inhibition is also consistent with the observation that the spindle checkpoint is active in the presence of VTT-006 as complete depletion of Ndc80 has been reported to abrogate the spindle checkpoint [6, 53]. Moreover, our results indicate that Aurora B accumulates at higher levels at kinetochores of unaligned chromosomes in VTT-006 treated cells, which could be related to amplification of the spindle checkpoint signal to maintain mitotic arrest, or to error correction via increased phosphorylation of Hec1 or other substrates at the centromeres and kinetochores of unaligned chromosomes [54].

Our data indicate that the compound has an ability to modulate the Ndc80-microtubule interaction *in vitro*. Live cell imaging and immunofluorescence assays showed that VTT-006 treated cells exhibited partial chromosome misalignment with cold stable kinetochore-microtubule attachments and reduced inter-kinetochore tension. These results are consistent with the study by Tooley et al. reporting chromosome misalignment, reduced inter-kinetochore tension and mitotic arrest in cells with mutations in Hec1 CHD at Lys residues 89, 115 and 116 [9], and the study by Sundin et al. reporting chromosome misalignment along with cold stable microtubules and reduced inter-kinetochore tension in cells with mutations in Hec1 binding partner Nuf2 CHD [34]. Thus, treatment of cells with VTT-006 produced a similar phenotype as mutation of key microtubule binding residues in the two CHDs of the Ndc80 complex. Lastly, chromosome movements including metaphase oscillations were clearly dampened in VTT-006 treated cells. Switches in chromosome movements originate from changes in kinetochore microtubule dynamics and kinetochore tension [55]. Interestingly, Hec1 has been reported to directly regulate microtubule dynamics [52]. In addition to this, the phosphorylation status of the tail region of Hec1 was shown to correlate with the amplitude of oscillation [56]. Together these data imply that the reduced metaphase plate oscillations observed in VTT-006 treated cells result from compound induced alterations in kinetochore microtubule dynamics and/or tension.

According to the analysis of time-lapse filmed cells, initial chromosome capture and lateral sliding along a microtubule soon after NEB appeared to be intact, which is in line with earlier findings showing Ndc80 is not required for lateral connections, which are thought to be mediated by dynein and Cenp-E [30, 32]. Moreover, all chromosomes in VTT-006 treated cells were capable of establishing connections with spindle microtubules as they exhibited movements in the vicinity of spindle poles or migrated to the metaphase plate. Importantly, the addition of VTT-006 on MG132 pre-treated metaphase cells led to a rapid impairment of metaphase chromosome alignment, which suggests that the defect in chromosome attachments also compromised the ability of cells to maintain amphitelic chromosome attachments. The extent of chromosome misalignment varied between individual cells and chromosomes within a cell. Some cells exhibited a few unaligned chromosomes while other chromosomes congressed to the metaphase plate. In some cells, most or all chromosomes remained unaligned after NEB until the cells died several hours later. At the moment we have no evidence-based explanation why some chromosomes are more sensitive to VTT-006 than others. This could reflect variability in the attachment dynamics between individual chromosomes to microtubules making them more or less vulnerable to VTT-006. Alternatively, there can be differences in the pharmacokinetics of VTT-006 at the level of individual kinetochores. For example, the accessibility or binding kinetics of the drug to its binding pocket may vary due to conformational differences between individual Ndc80 complexes. We cannot rule out the possibility that the compound has also other targets, such as microtubules, that can contribute to the phenotype we observe. Future research is warranted to examine the effects of VTT-006 in the presence of mutated Hec1 to evaluate the specificity of the compound.

In summary, we propose that VTT-006 impairs normal kinetochore-microtubule interactions by causing intermittent defects in the attachment status of individual kinetochores. In early mitotic cells, this results in two phenomena depending on initial chromosome attachment; chromosomes that instantly achieve amphitelic attachments after NEB can congress, albeit with sluggish dynamics, to the metaphase plate whereas chromosomes that end up with syntelic attachments after NEB remain near the poles due to defects in the syntelic-to-amphitelic conversion process. However, our findings that addition of VTT-006 to metaphase cells causes loss of alignment would seemingly contradict this model. Possibly in cells arrested at metaphase, the sudden influx of VTT-006 may weaken the strength of normal amphitelic attachments by causing release of individual microtubules at the kinetochore-microtubule interface eventually leading to loss of bi-polar attachment and metaphase chromosome alignment. The two phenomena can also be explained by additional mitotic targets of the compound.

Cell growth suppressive effects of VTT-006 occurred at the micromolar range (EC_50_ varied between 4.8 and 21.3 μM). Similarly, in the organotypic 3D culture of breast cancer cells, spheroid growth became limited at 5 μM level. From a therapeutic perspective, this indicates a need for further chemical optimisation of VTT-006 to achieve enhanced dose response without losing the demonstrated biological activity of the compound. Furthermore, future research should investigate in more detail the increased sensitivity to taxol induced by VTT-006 and extend the studies to several cell lines. This is important for evaluating the possible advantage of combination treatment in taxol-resistant cells. Also, the absorption, distribution, metabolism, and excretion (ADME) properties of the compound must be improved prior to testing with tumour xenografts in mice. However, as is the compound will provide a useful experimental tool to inhibit Ndc80 complex function for cell and cancer biologists who are investigating mitotic processes.

## MATERIALS AND METHODS

### Cell culture and reagents

Growth conditions for the cell lines are described in Supplementary Materials and Methods. Nocodazole (Sigma, M1404), Taxol (Paclitaxel, Sigma, T7191), ZM447439 (Tocris Bioscience, 2458), and MG132 (Sigma, C2211) were used in the experiments at 0.5, 0.1, 5, and 20 μM concentrations, respectively, unless indicated otherwise. Monastrol (Sigma, M8515) was used at 100 μM concentration and staurosporine (Sigma, S5921) at 1 μM concentration.

### Virtual screen

Chemical vendor libraries of small molecules from Chembridge, Enamine, IBS, VitasM, Specs, LifeChem, Asinex and ChemDiv with pre-calculated 3D conformations were docked into the crystal structure of Ndc80 (PDB ID: 2IGP) [10]. Small molecules containing a nitro group and a molecular weight greater than 500 Dalton were excluded. The protein crystal structure was pre-treated in Sybyl (Tripos, L.P. St. Louis, MO, USA) by removing all water molecules, and adding hydrogen atoms to the protein in an orientation optimal for hydrogen bonding. Docking was performed with docking software FRED [26] (OpenEye Scientific Software Inc., Santa Fe, NM, USA). Each small molecule was docked with FRED’s native function followed by re-scoring with five additional scoring functions: shapegauss, plp, chemgauss3, chemscore and screenscore. Each docking score was transformed into a Z-score by substracting the mean and dividing by the standard deviation of all docking scores of the respective scoring function. For the final hit list, molecules were taken that had a Z-score smaller than minus three with at least three scoring functions. These were still subjected to a visual inspection where improbable docking poses were excluded. In the end, we had 138 small molecules that were ordered from chemical suppliers. In addition to FRED software, VTT-006 docking was performed with Glide/Maestro package (version 9.6, Schrödinger Inc, Portland, OR, USA), which was used to create image in Figure 1C. Molecular Operating Environment (MOE) (2013.08; Chemical Computing Group Inc., Montreal, Canada) was used to create interaction maps (Figure 1D and Supplementary Figure 1).

### Cell based screen

HeLa H2B-GFP [57] cells were plated on 384-well plates with a Multidrop Combi (Thermo Fisher Scientific, Waltham, MA, USA) and selected compounds from the virtual screen were applied with Hamilton Microlab Star robotics (Hamilton, Reno, NV, USA) on cells, 24 h after seeding, in 3 different concentrations, 0.2, 2 and 20 μM. Taxol was used as a positive control as a mitotic arrest inducing compound at 3 μM concentration and DMSO as negative control. The plates were imaged 18 and 42 h after compound addition using transmitted light and filter setting Alexa 488 to visualize GFP. Wells that had mitotic arrest were scored as positive hits.

### Anisotropy

Fluorescence measurements were made on a QuantaMaster-1-spectrofluorometer operated in T-state (Photon Technology International, Lawrenceville, GA, USA). To determine excitation and emission properties of the compounds of interest excitation and emission spectra were collected at different wavelengths in PBS at 23^°^C. The binding of the compounds to proteins was determined by measuring steady state anisotropy of the compound, and anisotropy in the presence of varying concentrations of Bonsai or Aurora B in PBS. Anisotropy measurements for VTT-006 were acquired using 350 nm for excitation and 450 nm for emission. The dissociation constant (K_d_) was calculated using a one site binding model that accounts for ligand depletion as previously described [58, 59]. The data analysis and fitting was done with the Microcal Origin 7.5 software (Origin Lab, Northampton, MA, USA).

### Recombinant protein production

Ndc80^bonsai^ (referred to as Bonsai) was a kind gift from A. Musacchio [11]. For anisotropy, Bonsai was purified as described below. Bonsai expression in *E. coli* BL21(DE3) was induced with 500 μM IPTG at OD_600_ = 0.45–0.7 for 16 h at RT. Cells were harvested by centrifugation at 3500 g. The bacterial pellet was resuspended in lysis buffer (50 mM Tris-HCl, pH 7.6, 300 mM NaCl, 1 mM DTT, 1 mM EDTA, Complete Protease Inhibitor Cocktail Tablets from Roche) and sonicated. After this, lysate was cleared by centrifugation at 50 000 × g for 60 min. Supernatant was filtered and Bonsai purified using ÄKTA prime plus system (GE Healthcare, Waukesha, WI, USA) and GST Sepharose Fast Flow (GE Healthcare). Cleavage buffer (50 mM Tris-HCl, pH 7.6, 150 mM NaCl, 1 mM DTT, 1 mM EDTA) was used for equilibration and elution of Bonsai. PreScission protease (GE Healthcare) was applied into the column and left overnight at +4°C to cleave off GST followed by elution of Bonsai. Fractions containing Bonsai were collected and used for anisotropy. For GST-Aurora B production, BaculoGold^™^ expression system (BD Biosciences) was used to express Aurora B in Sf9 insect cells as described previously [60].

### TIRF microscopy

Total internal reflection fluorescence (TIRF) microscopy was performed on a custom illumination system as previously described [52, 61, 62]. Recombinant GFP-tagged Ndc80 complex used for TIRF was expressed and purified as previously described [52, 63]. Taxol-stabilized microtubules (1% Alexa-568-labeled) were bound to coverslips using a “rigor” kinesin [64]. GFP-tagged Ndc80 complex was assayed at 6–10 pM in BRB80 (80 mM Pipes, 120 mM K^+^, 1 mM MgCl_2_, and 1 mM EGTA, pH 6.9) with 8 mg/mL BSA, 10 μM Taxol, and an oxygen scavenger system (200 μg/mL glucose oxidase, 35 μg/mL catalase, 25 mM glucose, and 5 mM DTT). VTT-006 was assayed at 10 μM, and DMSO was used as a control. Analysis of TIRF microscopy data was performed using custom software (available upon request) in LabVIEW (National Instruments, Austin, TX, USA) and Igor Pro (Wavemetrics, Tigard, OR, USA), as previously described [61].

### Immunofluorescence

For immunofluorescence, cells growing on coverslips were fixed for 15 min in 60 mM Pipes, pH 6.9, 25 mM Hepes, 10 mM EGTA, 4 mM MgSO4 (PHEM) containing 2% paraformaldehyde, 0.2% glutaraldehyde (added for tubulin staining), and 0.5% Triton X-100. The coverslips were rinsed in 10 mM MOPS, pH 7.4, 150 mM NaCl, 0.05% Tween-20 (MBST), and blocked for 1 h in 20% boiled normal goat serum (bngs) in MBST. Cells were stained for 1 h at RT or overnight at +4°C with primary antibodies against Aurora B (AIM-1, BD, 611083), phospho-Thr232 (Rockland 600-401-677), Bub1 (Upstate, 05-899), BuBR1 (Abcam, ab4637), Hec1 (Abcam, ab3613), pericentrin (Abcam, ab4448), tubulin (Abcam, ab7291) or human autoimmune serum (Crest, Antibodies Incorporated) followed by Alexa Fluor 488, 555 and 647 dyes against mouse, rabbit and human antigens (Invitrogen) diluted in MBST. DNA was counter stained with DAPI before mounting in Vectashield (Vector laboratories, H-1000). The cold-Ca^++^ assay to analyze the stability of kinetochore-microtubule attachment was performed by placing coverslips in ice cold lysis buffer (0.1 M Pipes, pH 6.95, 80 μM CaCl_2_, 1% Triton X-100) for 5 minutes followed by fixation for tubulin staining.

### Microscopy

ScanR high content imager (Olympus Corporation, Tokyo, Japan) equipped with a Hamamatsu ORCA-ER CCD digital camera (Hamamatsu Photonics, Hamamatsu City, Japan) was used to analyze cell-based screen results. Live cell phase-contrast imaging was performed with an IncuCyte live-cell imager (Essen Instruments Ltd., Hertfordshire, UK). A Zeiss inverted 200M microscope (Zeiss GmbH, Jena, Germany) equipped with a Hamamatsu ORCA-ER camera and MetaMorph software (Molecular Devices, Downingtown, USA) was used for analysis of kinetochore protein levels. Kinetochore intensities were quantified from maximum projections created from a Z-stack of images acquired every 0.5 μm. Zeiss Axiovert 200M microscope equipped with spinning disk CSU22 confocal scanner (Yokogawa, Tokyo, Japan) and SlideBook 5.0 software (Intelligent Imaging Innovations, Inc. Denver, CO, USA) was used for acquiring images of 3D cultures, for the analysis of kinetochore distances and live-cell imaging of Hela H2B-GFP and GFP-Spc24 HeLas with environmental control.

### Western blotting

HeLa cells were centrifuged and washed once with cold PBS before preparation of extract or freezing the pellets in liquid nitrogen. For the preparation of extracts, cells were lysed in 20 mM Tris-HCl (pH 7.7), 100 mM KCl, 50 mM sucrose, 1 mM MgCl_2_, 0.1 mM CaCl_2_, 0.5% TX-100 (APC-buffer) containing protease inhibitor cocktail (Roche, 04693132001) and phosphatase inhibitor PhosSTOP (Roche, 4906837001) for 7 min on ice, and cell lysates were cleared by centrifugation. Equal amounts of samples were loaded and run on 4–20% gradient gels followed by semi-dry transfer with Trans-blot (Bio-Rad, Hercules, CA). Membranes were blocked in 5% milk or Odyssey blocking buffer (Fisher Scientific, NC9877369) in TBS for 1 h, followed by primary antibody incubation for 1 h at RT or overnight at +4°C in TBST (TBS containing 0.05% Tween), and secondary antibody for 1 h at RT in TBST. Primary antibodies against Hec1 (Abcam, ab3613), cleaved PARP (Cell Signaling, 9546, dilution) and GAPDH (Advanced ImmunoChemical Inc., mAb 6C5) were followed by secondary antibody Alexa Fluor^®^ anti-mouse 680 (Invitrogen). Signals were detected using Odyssey Infrared Imaging System (LI-COR Biosciences, Lincoln, NE, USA).

### *In vitro* tubulin polymerization assay

Fluorescence *in vitro* tubulin polymerization assay (Cytoskeleton Inc., BK011) was performed according to the manufacturer’s instructions. The reaction contained PEM buffer, glycerol (13.8%), GTP (1 mM), porcine brain >99% pure tubulin (2 mg/ml), fluorescent reporter (5 μM), and test compounds. Taxol (3 μM), vinblastine (3 μM) and DMSO were used as controls. Tubulin polymerization reaction was measured at 1 min intervals for 60 min at 37°C with excitation at 355 nm and emission at 460 nm with Victor 1420 Multilabel HTS Counter (Perkin Elmer, Waltham, MA, USA).

### 3D organotypic cell culture

3D cell culture was performed with some modifications to the previously described protocol [40]. A bottom gel (4 mg/ml) was prepared with growth factor reduced and phenol-red free Matrigel (BD Biosciences, 356231) in RPMI 1640 and pipetted to the wells of tissue culture treated Angiogenesis 96-well plates (Ibidi Gmbh, Planegg/Martinsried, Germany, cat. no 89646). Plates were centrifuged for 20 min at 200 rpm and allowed to polymerize at 37°C for 1 h. Cells were collected by trypsin and mixed with Matrigel (2 mg/ml) in growth medium and applied to wells with cell density of 1000–1500 cells/well. This upper gel containing cells was allowed to polymerize for 3 h to overnight at 37°C before medium was added to wells. Growth media recipes are described in Supplementary Materials and Methods. MCF7 cells were grown until day 6 before DMSO, VTT-006 or Taxol were added and cells were grown in the presence of the compounds for 6 days. MDA-MB-231 SA cells were incubated with compounds for 5 days starting at day 2. Drugs were applied to cells at different time points when spheroid size was optimal and fresh medium with drugs was changed every 2–3 days. Treatments were performed in triplicate.

### 3D image acquisition and analysis

Multicellular structures were double-stained with Calcein-AM fluorescent dye (Molecular Probes, C1430) and Ethidium homodimer-1 (Molecular Probes, E1169). 3D confocal images were acquired using Zeiss Plan-Neofluar 5x objective. Maximum intensity projections were created and background noise was removed by normalization with SlideBook. Images were analyzed using VTT’s in-house developed proprietary software AMIDA, software specifically designed for multicellular structure segmentation and measurement of biologically important morphological features such as size, roundness and cellular invasiveness [65]. All statistical analyses and plotting of numerical data (post-image analysis) were performed using R, an open source programming language and software environment for statistical computing and graphics (http://cran.r-project.org).

### Measurement of EC_50_ values

Cells were plated on 384-well plates with Multidrop Combi (Thermo Scientific, Waltham, USA). VTT-006 was added to cells 24 h later at 7 different concentrations between 1 nM-20 μM in 6 replicate wells per concentration. CellTiter Glo reagent (Promega) was added to cells following manufacturer’s protocol at 48 or 72 h and cell viability was analyzed with Envision 2100 multilabel plate reader (Perkin Elmer, Waltham, MA, USA) by measuring luminescence. Data analysis was performed with Graph Pad Prism 4 (GraphPad Software, Inc, La Jolla, CA, USA). EC_50_ values were determined from 3 replicate experiments.

## SUPPLEMENTARY MATERIALS













## Abbreviations

ADME: Absorption, Distribution, Metabolism, And Excretion
Bngs: Boiled normal goat serum
BSA: Bovine Serum Albumin
CHD: Calponin Homology Domain
DAPI: 4’,6-diamidino-2-phenylindole
DMSO: dimethyl sulfoxide
DNA: deoxyribonucleic acid
DTT: dithiothreitol
GAPDH: glyceraldehyde-3-phosphate dehydrogenase
GFP: Green Fluorescent Protein
Hec1: highly expressed in cancer 1
HTS: high-throughput screen
MOE: Molecular Operating Environment
NEB: Nuclear Envelope Breakdown
PARP: poly (ADP-ribose) polymerase
PBS: Phosphate Buffered Saline
RNA: ribonucleic acid
RNAi: RNA interference
siRNA: small interfering RNA
TIRF: Total Internal Reflection Fluorescence
