# Supplementary material for: VTT-006, an anti-mitotic compound, binds to the Ndc80 complex and suppresses cancer cell growth in vitro
Source: Oncoscience. 2021 Dec 10;8:134–53. doi: 10.18632/oncoscience.549 (PMC8667816; doi:10.18632/oncoscience.549)
Supplement: Supplementary file 1 [file oncoscience-08-549-s001.pdf]

VTT-006, an anti-mitotic compound, binds to the Ndc80 complex and suppresses cancer cell growth *in vitro*

SUPPLEMENTARY MATERIAL

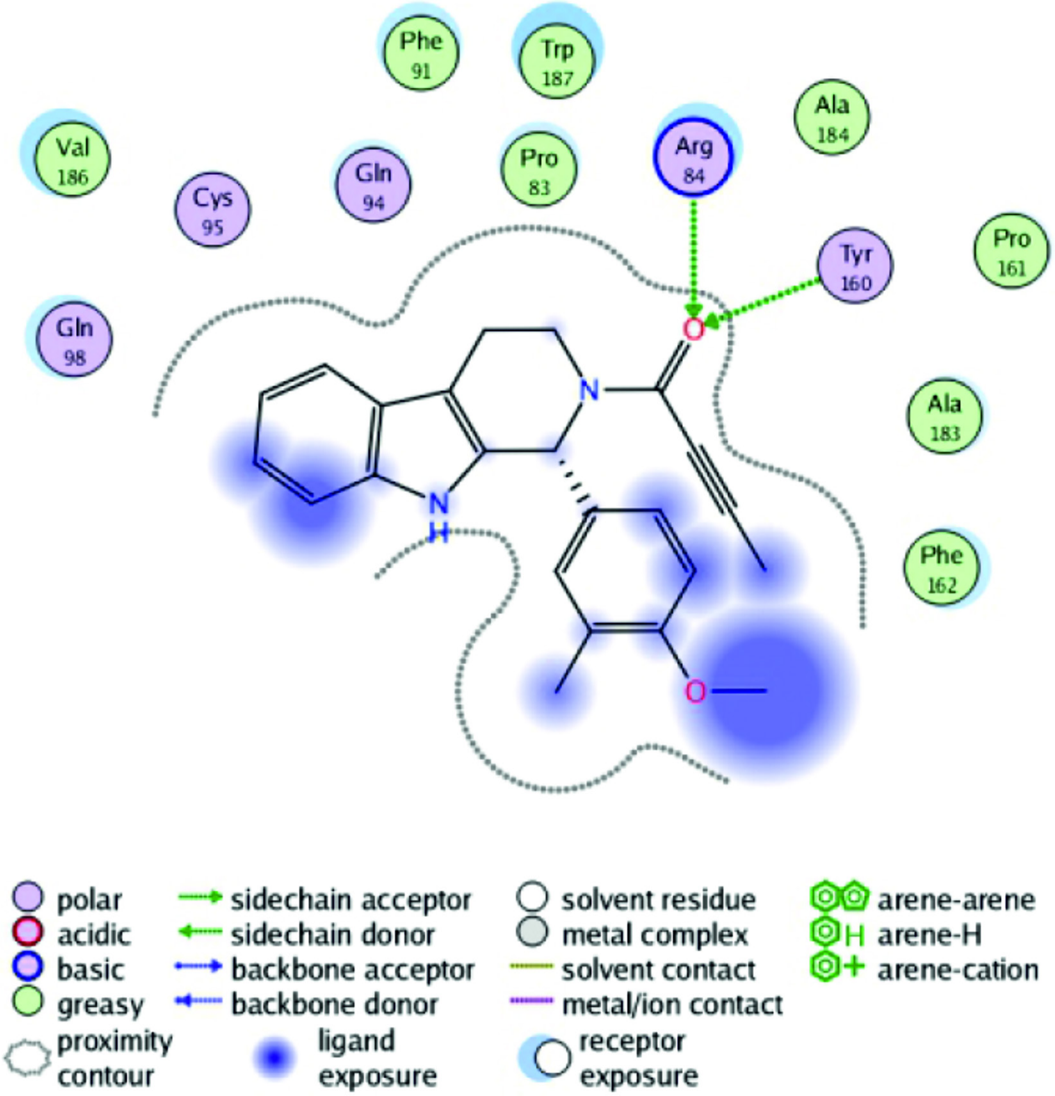

Supplementary Figure 1: Predicted interactions between VTT-006 and Hec1 CH domain. Various amino acid residues located in the Hec1 CH domain are shown. VTT-006 is predicted to interact via H-bonds between carbonyl oxygen of VTT-006 and both Arg84 and Tyr160 of Hec1. In addition, the structure of VTT-006 is proposed to have favourable vdW-interactions with the lipophilic residues of the binding cavity. Image was created with Molecular Operating Environment (MOE).

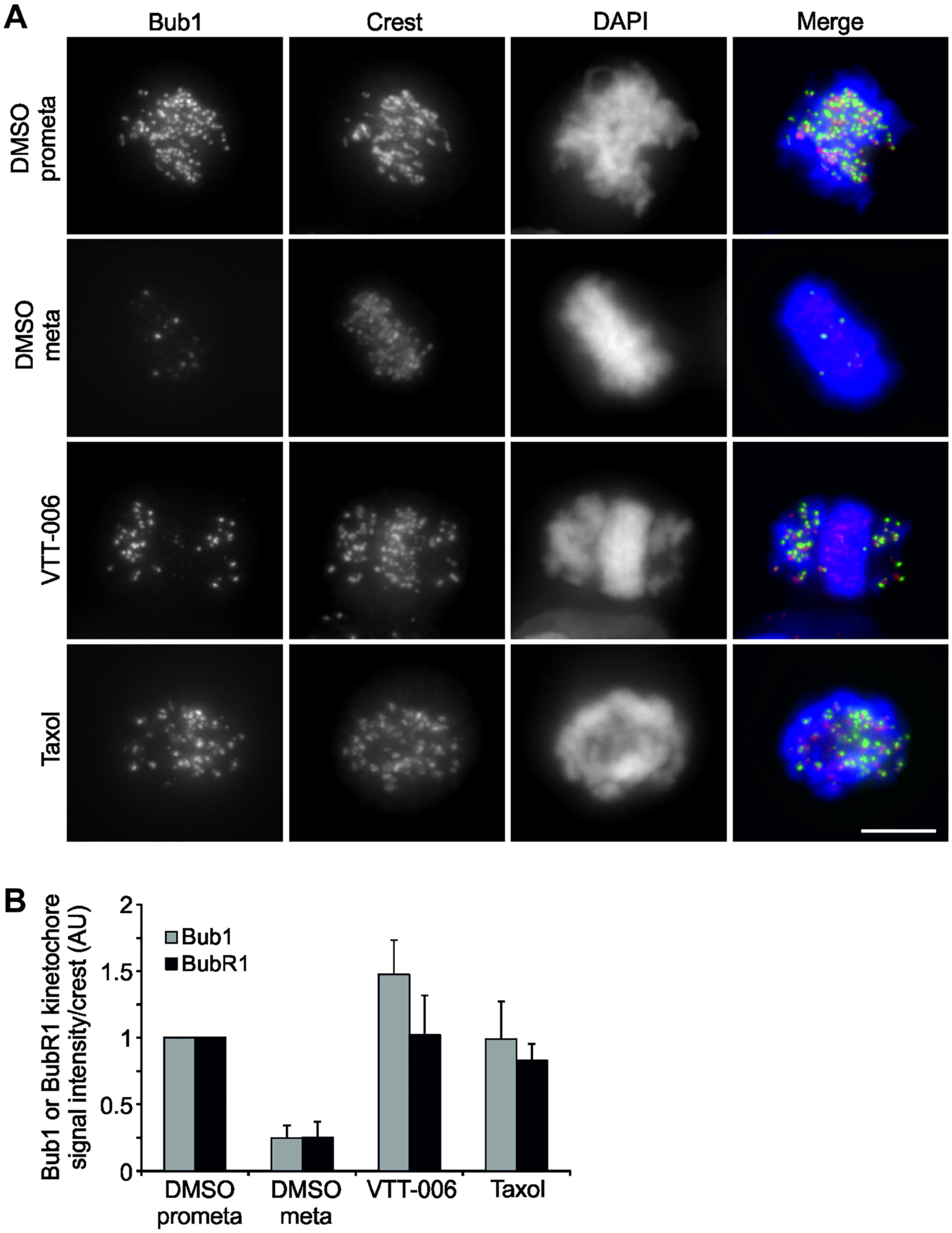

Supplementary Figure 2: Mitotic checkpoint is active in VTT-006 treated cells. HeLa cells were treated with DMSO, 10 μM VTT-006 or 100 nM Taxol for 6 h, fixed and stained with anti-Bub1, anti-BubR1 and crest antibodies. (**A**) Images from Bub1 staining showing clear signals at the kinetochores of unaligned chromosomes in VTT-006 treated cells indicating continued spindle checkpoint activity. Merge shows Bub1 (green), crest (red) and DAPI (blue). (**B**) Quantification of Bub1 and BubR1 kinetochore signals. For VTT-006, only unaligned chromosomes near spindle poles were quantified. Result is average ± SD from 3 replicate experiments (8–15 cells and 20 kinetochores per cell were quantified in each experiment). Scale bar = 10 μm.

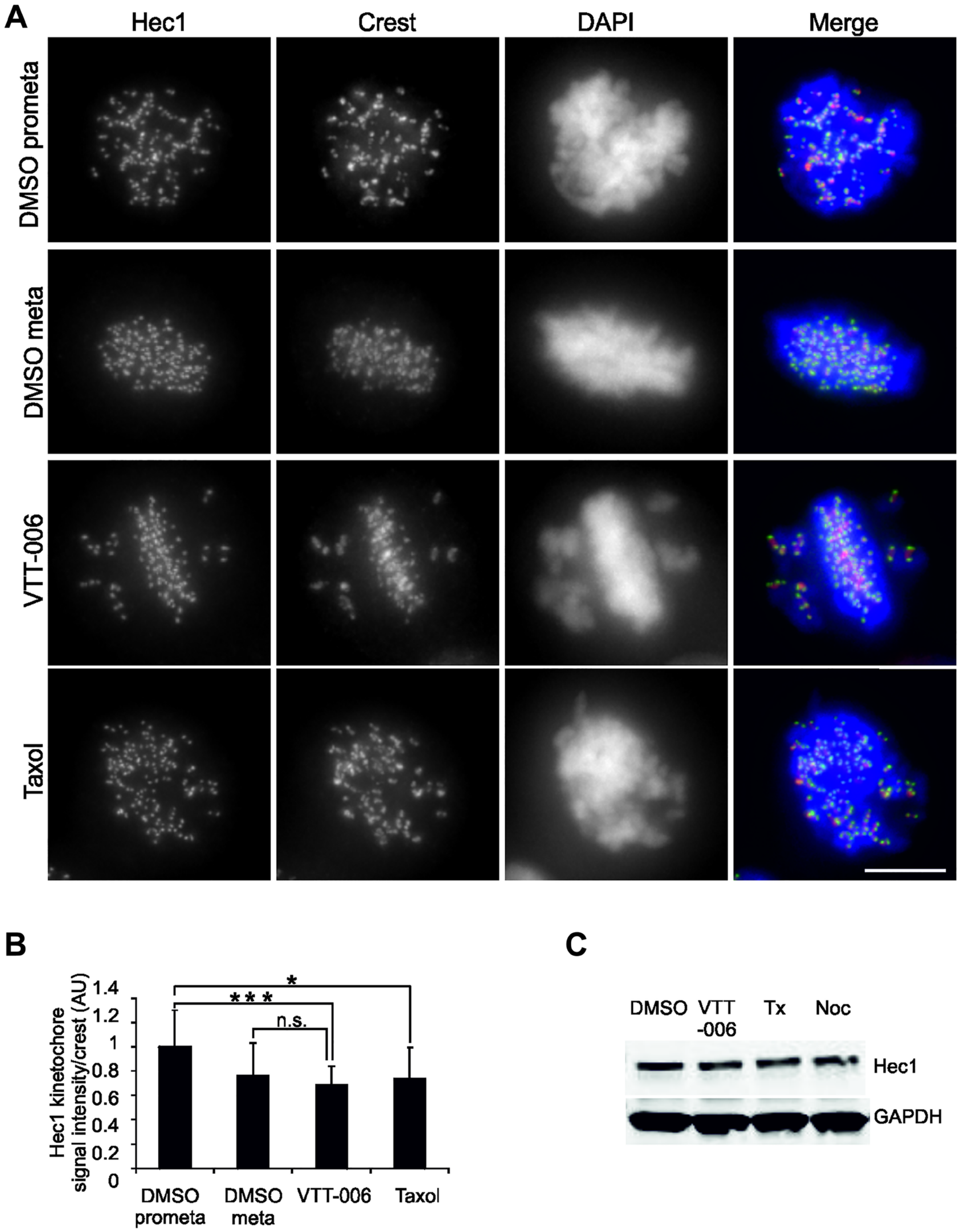

Supplementary Figure 3: Analysis of Hec1 kinetochore level and total protein level in VTT-006 treated cells. (**A**) Images of HeLa cells stained with Hec1 antibody (green in merge) and crest (red). (**B**) Quantification of Hec1 kinetochore level. Average of 15 cells (300 randomly selected individual kinetochores) ± SD is shown. (**C**) Western blot showing Hec1 and GAPDH after treatment with indicated compounds. HeLa cells were treated with DMSO, 10 µM VTT-006 or 100 nM Taxol for 12 h before fixing for immunofluorescence or collecting cells for Western blot. Scale bar = 10 µm.

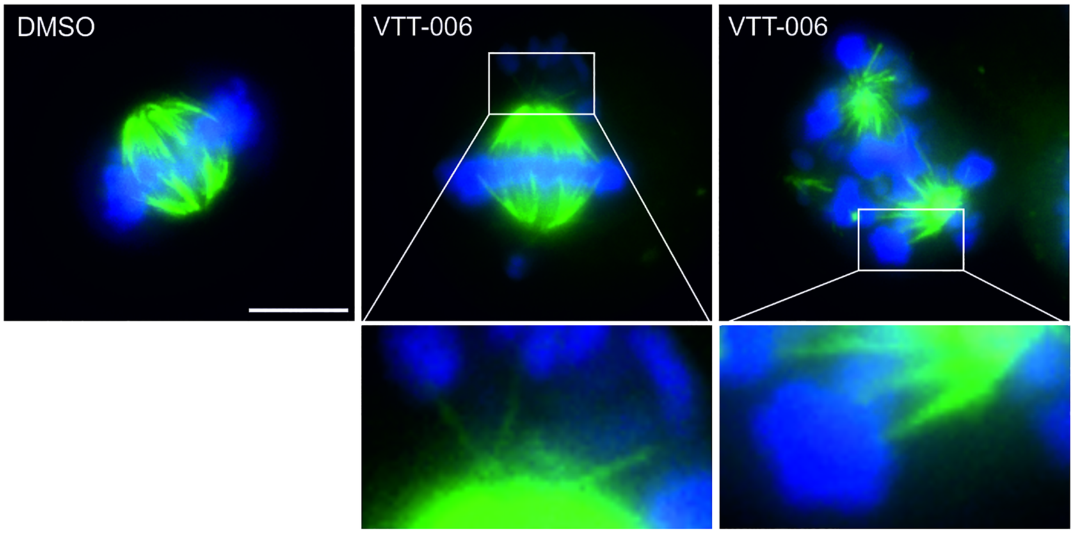

Supplementary Figure 4: Kinetochore-microtubule attachments are stable in VTT-006 treated cells. Overlays showing Hela cells treated with DMSO or 5 μM VTT-006 for 4 h, lysed in cold Ca^{++} buffer and stained for tubulin (green) and DNA (blue). Insets show high magnification views of microtubules extending towards unaligned chromosomes. Scale bar = 10 μm.

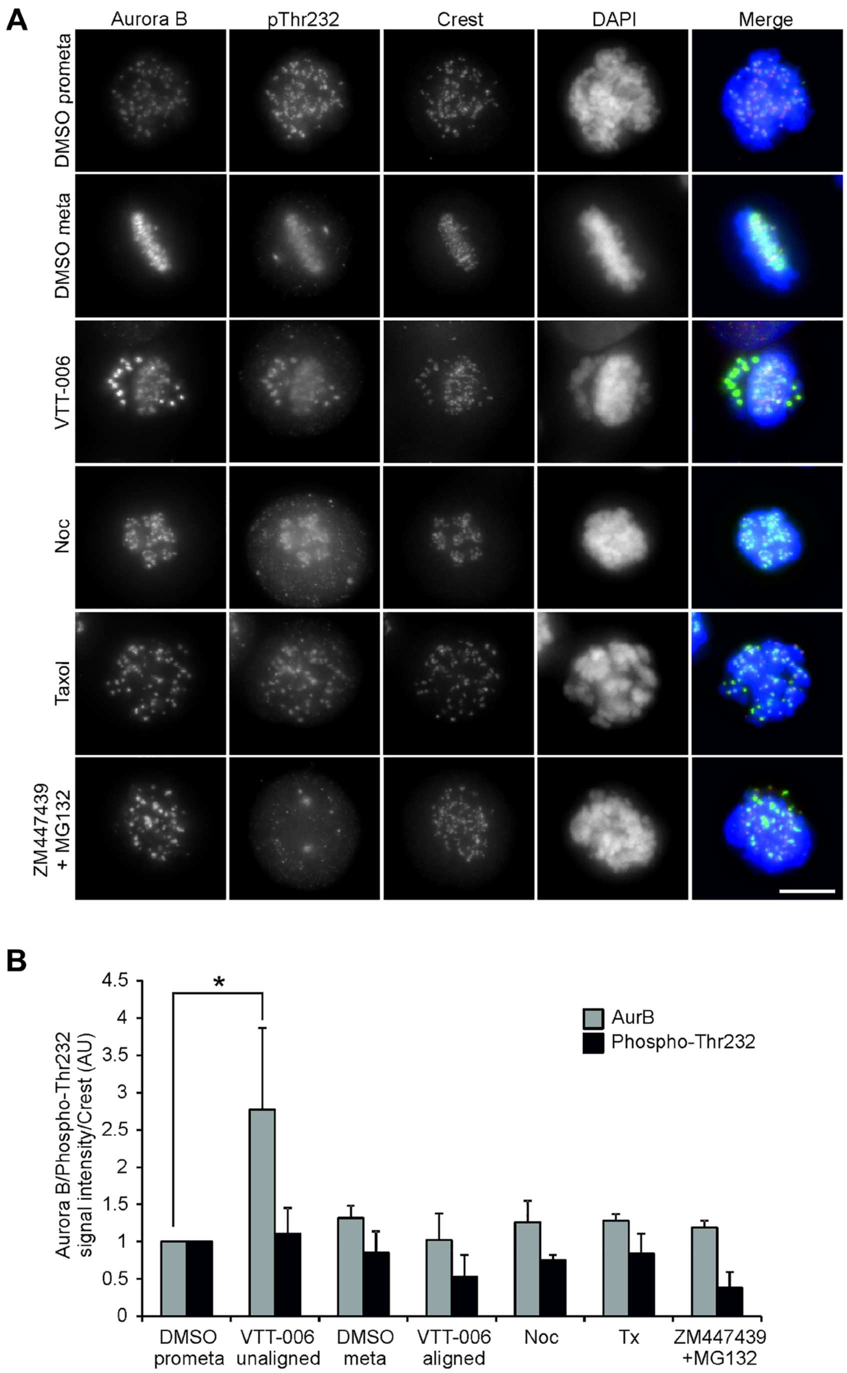

Supplementary Figure 5: VTT-006 increases Aurora B accumulation at centromeres of unaligned chromosomes. (**A**) Immunofluorescent images of cells treated with indicated compounds showing Aurora B accumulation at centromeres of unaligned chromosomes. (**B**) Quantification of Aurora B and active Aurora B (phospho-Thr232) from centromere/kinetochore region. Result is average from 3 replicate experiments ± SD. 20 kinetochores were quantified from 5–10 cells in each experiment. HeLa cells were treated for 6 h with DMSO, 10 µM VTT-006, 500 nM nocodazole, 100 nM Taxol or 5 µM ZM447439 and 20 µM MG132 followed by staining with anti-Aurora B, phospho-Thr232 and crest antibodies and DAPI. Aurora B (green), crest (red) and DAPI (blue) are shown in merge image. Star (*) denotes statistical significance, $p \leq 0.05$. Scale bar = 10 µm.

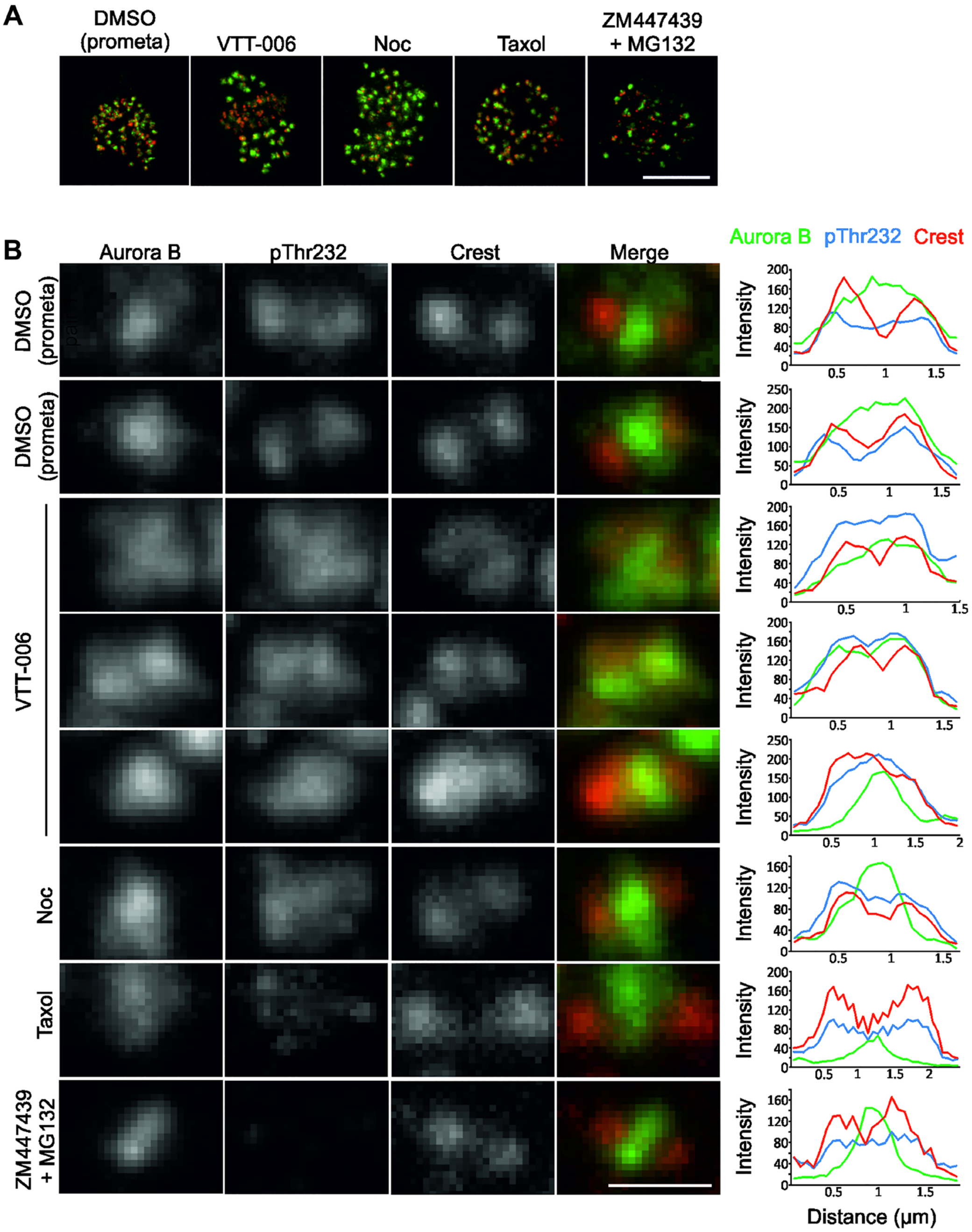

Supplementary Figure 6: Fine localization of Aurora B and active Aurora B (phospho-Thr232) in VTT-006 treated cells. (**A**) Merge images showing Aurora B (green) and crest (red) in cells treated with indicated compounds. (**B**) Close-up images of example kinetochore pairs selected from images in panel A. Images were acquired with a 100× objective using a confocal microscope. Line scans were prepared in Metamorph. For clarity, merge shows only Aurora B (green) and crest (red). HeLa cells were treated for 6 h with DMSO, 10 µM VTT-006, 500 nM nocodazole, 100 nM Taxol or 5 µM ZM447439 and 20 µM MG132 followed by staining with anti-Aurora B, phospho-Thr232 and crest antibodies and DAPI. Scale bar = 10 µm in A and 1 µm in B.

Supplementary Movie 1: VTT-006 causes chromosome misalignment, mitotic arrest and aberrant cell division. See Supplementary Movie 1

Supplementary Movie 2: Control cells undergoing cell division. See Supplementary Movie 2

Supplementary Movie 3. MG132 treated cells remain arrested at metaphase. See Supplementary Movie 3

Supplementary Movie 4: VTT-006 disrupts metaphase plate alignment and causes chromosome movement towards spindle poles. See Supplementary Movie 4

Supplementary Movie 5: Taxol severely disrupts metaphase chromosome alignment. See Supplementary Movie 5

SUPPLEMENTARY MATERIALS AND METHODS

Cell culture

Cell line	Description	Growth medium	Source and year when cells were obtained
HeLa	cervical adenocarcinoma	DMEM (high glucose), 10% FBS, 20 mM HEPES, 0.1 mM non-essential amino acids, 1 mM sodium pyruvate, 2 mM L-glutamine, 0.1 mg/ml penicillin/streptomycin	Prof. Gary Gorbsky, Oklahoma Medical Research Foundation, Oklahoma City, USA, 2004
HeLa-H2B-GFP	HeLa cells stably expressing H2B-GFP	DMEM (high glucose), 10% FBS, 20 mM HEPES, 0.1 mM non-essential amino acids, 1 mM sodium pyruvate, 2 mM L-glutamine, 0.1 mg/ml penicillin/streptomycin, 2 µg/ml blasticidin	[57], 2006
GFP-Spc24-HeLa	HeLa cells stably expressing GFP-Spc24	DMEM (high glucose), 10% FBS, 20 mM HEPES, 0.1 mM non-essential amino acids, 1 mM sodium pyruvate, 2 mM L-glutamine, 0.1 mg/ml penicillin/streptomycin, 200 µg/ml G418	Prof. Todd Stukenberg, University of Virginia, Charlottesville, USA, 2004
MCF10A	mammary epithelial cells	1:1 DMEM: HAM's F12, 5 % horse serum, 2 mM L-glutamine, 10 ug/ml insulin, 5 ug/ml hydrocortisone, 20 ng/ml EGF, 100 ng/ml cholera toxin	ATCC CRL-10317, 2006
MCF7	breast adenocarcinoma	DMEM (low glucose), 10% FBS, 2 mM L-glutamine, 0.1 mg/ml penicillin/streptomycin	ICLC HTL95021, 2004
MDA-MB-231	breast adenocarcinoma	DMEM (high glucose), 10% FBS, 0.1 mM non-essential amino acids, 2 mM L-glutamine, and 0.1 mg/ml penicillin/streptomycin	ATCC HTB-26, 2004
MDA-MB-231 SA	breast adenocarcinoma	DMEM (high glucose), 10% FBS, 0.1 mM non-essential amino acids, 2 mM L-glutamine, and 0.1 mg/ml penicillin/streptomycin	Prof. Theresa A. Guise, University of Virginia, Charlottesville, USA, 2004
LNCap	prostatic adenocarcinoma	RPMI 1640 supplemented with 10% FBS, 2 mM L-glutamine, and 0.1 mg/ml penicillin/streptomycin	ATCC CRL-1740, 2003
RWPE-1	prostate epithelial cells	KSFM containing BPE and EGF (Gibco 17005-042)	ATCC CRL-11609, 2006
RWPE-2-W99	tumorigenic derivative of RWPE-1	KSFM containing BPE and EGF (Gibco 17005-042)	ATCC CRL-2853, 2006
Ep156T	prostate epithelial cells	KSFM containing BPE and EGF (Gibco 17005-042)	Prof. Varda Rotter, Weitzmann Institute, Rehovot, Israel, 2006
HCT116	colorectal carcinoma	McCoy's 5A, 10% FBS, 2 mM L-glutamine, 0.1 mg/ml penicillin/streptomycin	Prof. Lauri Aaltonen, University of Helsinki, Helsinki, Finland, 2009
A549	lung adenocarcinoma	RPMI 1640 supplemented with 10% FBS, 2 mM L-glutamine, and 0.1 mg/ml penicillin/streptomycin	ATCC CCL-185, 2005
Ovcar-3	ovary adenocarcinoma	RPMI 1640 supplemented with 10% FBS, 2 mM L-glutamine, and 0.1 mg/ml penicillin/streptomycin	[1], 2005

Statistics

Statistical analysis was performed with student's *t*-test.

SUPPLEMENTARY REFERENCE

1. Hamilton TC, Young RC, McKoy WM, Grotzinger KR, Green JA, Whang-Peng J, Rogan AM, Ozols RF, Chu EW, Green WR. Characterization of a Human Ovarian Carcinoma Cell Line (NIH:OVCAR-3) with Androgen and Estrogen Receptors. Cancer Res. 1983; 43:5379–89. PMID:6604576
